# Development of an Inflammatory CD14^+^ Dendritic Cell Subset in Humanized Mice

**DOI:** 10.3389/fimmu.2021.643040

**Published:** 2021-03-15

**Authors:** Ryutaro Iwabuchi, Keigo Ide, Kazutaka Terahara, Ryota Wagatsuma, Rieko Iwaki, Hiroko Matsunaga, Yasuko Tsunetsugu-Yokota, Haruko Takeyama, Yoshimasa Takahashi

**Affiliations:** ^1^Department of Immunology, National Institute of Infectious Diseases, Tokyo, Japan; ^2^Department of Life Science and Medical Bioscience, Waseda University, Tokyo, Japan; ^3^Computational Bio Big-Data Open Innovation Laboratory, National Institute of Advanced Industrial Science and Technology, Tokyo, Japan; ^4^Research Organization for Nano and Life Innovation, Waseda University, Tokyo, Japan; ^5^Department of Medical Technology, School of Human Sciences, Tokyo University of Technology, Tokyo, Japan; ^6^Institute for Advanced Research of Biosystem Dynamics, Waseda Research Institute for Science and Engineering, Waseda University, Tokyo, Japan

**Keywords:** humanized mice, dendritic cell, DC3, CD14, inflammatory response, S100A8, S100A9

## Abstract

Humanized mouse models are attractive experimental models for analyzing the development and functions of human dendritic cells (DCs) *in vivo*. Although various types of DC subsets, including DC type 3 (DC3s), have been identified in humans, it remains unclear whether humanized mice can reproduce heterogeneous DC subsets. CD14, classically known as a monocyte/macrophage marker, is reported as an indicator of DC3s. We previously observed that some CD14^+^ myeloid cells expressed CD1c, a pan marker for *bona fide* conventional DC2 (cDC2s), in humanized mouse models in which human *FLT3L* and *GM-CSF* genes were transiently expressed using *in vivo* transfection (IVT). Here, we aimed to elucidate the identity of CD14^+^CD1c^+^ DC-like cells in humanized mouse models. We found that CD14^+^CD1c^+^ cells were phenotypically different from cDC2s; CD14^+^CD1c^+^ cells expressed CD163 but not CD5, whereas cDC2s expressed CD5 but not CD163. Furthermore, CD14^+^CD1c^+^ cells primed and polarized naïve CD4^+^ T cells toward IFN-γ^+^ Th1 cells more profoundly than cDC2s. Transcriptional analysis revealed that CD14^+^CD1c^+^ cells expressed several DC3-specific transcripts, such as CD163, S100A8, and S100A9, and were clearly segregated from cDC2s and monocytes. When lipopolysaccharide was administered to the humanized mice, the frequency of CD14^+^CD1c^+^ cells producing IL-6 and TNF-α was elevated, indicating a pro-inflammatory signature. Thus, humanized mice are able to sustain development of functional CD14^+^CD1c^+^ DCs, which are equivalent to DC3 subset observed in humans, and they could be useful for analyzing the development and function of DC3s *in vivo*.

## Introduction

Dendritic cells (DCs) are antigen-presenting cells essential for the innate and acquired immunity (1–3). They comprise various subpopulations that include not only conventional DCs type 1 (cDC1s), type 2 (cDC2s), and plasmacytoid DCs (pDCs) in the systemic compartment, but also monocyte-derived DCs (MoDCs) in the peripheral tissues. These DC subsets have been historically classified on the basis of their phenotype, functionality, and localization in both humans and mice (4–7). However, differences in phenotypes and functionalities exist in the same DC subsets between humans and mice (8–12). Furthermore, there is a new DC subset in humans, DC3s, whose equivalent counterparts have not yet been identified in mice (13). Thus, it is valuable to establish animal models that precisely reproduce human DC subsets, as they can prove useful for translational research where DCs are utilized for immunotherapy against cancer and infectious diseases (14–16).

Humanized mice, which are reconstituted with human immune cells, are an attractive model for studying differentiated human DCs *in vivo* (17–21). Widely used humanized mouse models have been constructed by transplanting hematopoietic stem cells (HSCs) into severely immunodeficient mice, such as non-obese diabetic (NOD)/SCID/IL2Rγ^null^ (NSG or NOG) mice (22, 23). However, they show poor human DC differentiation and maturation owing to a lack of the responsible cytokines (22, 24). To overcome this limitation, treatment with FMS-like tyrosine kinase 3 ligand (FLT3L) was introduced to improve human DC differentiation in humanized mice (8, 25–27). Recently, another humanized mouse model was established by transplantation of cytokine-expressing stromal cell lines, resulting in improved cDC differentiation (28). However, it remains unclear how precisely humanized mice can reproduce the heterogeneity of DC subsets in humans.

According to the established classification (5), cDCs are defined as distinct subsets from monocytes and macrophages because the DC subsets and monocytes/macrophages are derived from different progenitor cells. However, recent studies revealed that CD1c^+^ cDCs in human peripheral blood mononuclear cells (PBMCs) comprise heterogeneous subsets, including a subset with monocyte-like characteristics. This subset was originally reported by Villani et al. and named “DC3” (29). DC3s were separated from cDC2s based on the expression of monocyte-related genes, including *CD14, CD163, S100A8*, and *S100A9*, using unbiased transcriptional classification (29). DC3s can be isolated from human PBMCs using DC-related markers and CD14 and/or CD163, and their transcriptional profiles and functionalities are becoming clear (30, 31). In these studies (30, 31), key cytokines (FLT3L and GM-CSF) have been identified for *in vivo* differentiation of DC3s in humans (30) and PBMC-engrafted NSG mice (31). Moreover, the ontogeny and further transcriptional and functional characteristics of DC3s have been discovered using *IRF8* mutated bone marrow and blood samples in humans (32).

Following the report by Villani et al. (29), we previously observed the development of CD14^+^ DC-like cells along with *bona fide* cDC2s in CD1c^+^ population in lymphoid tissues of humanized mice (21). Therefore, we aimed to elucidate whether these CD14^+^CD1c^+^ cells were equivalent to DC3s and the humanized mice could be used for analyzing DC3 development. In this study, we investigated phenotype, functionality, and transcriptional profiles of CD14^+^CD1c^+^ cells compared to those of cDC2s.

## Materials and Methods

### Construction of Humanized Mice

Humanized mice were constructed as described previously (21, 33, 34) using NOD/SCID/Jak3^null^ (NOJ) mice which have an identical phenotype to NSG and NOG mice, with minor modifications. In brief, human HSCs were isolated from umbilical cord blood using the CD133 MicroBead Kit (Miltenyi Biotec, Bergisch Gladbach, Germany). The isolated HSCs (1–1.8 × 10^5^ cells) were transplanted into the livers of non-irradiated newborn NOJ mice (≤2 days old). Every 4 weeks, starting from 8 week after HSC transplantation, approximately 30 μl of peripheral blood was obtained from the facial vein to determine the extent of chimerism (the percentage of human CD45^+^ cells within the total peripheral blood cells). Individual humanized NOJ (hNOJ) mice used in this study are listed in Supplementary Table 1 with information, including the HSC donor ID number, age, and chimerism. Fifteen- to seventeen-week old hNOJ mice were used in this study. All mice were maintained under specific pathogen-free conditions in the animal facility at the National Institute of Infectious Diseases (NIID) (Tokyo, Japan).

### *In vivo* Transfection of Human FLT3L and GM-CSF Using Hydrodynamic Gene Delivery in hNOJ Mice

hNOJ mice at 15–16 weeks post-humanization were subjected to the *in vivo* transfection (IVT) with human *FLT3L* and *GM-CSF* genes to enhance DC development as described previously (21), with minor modifications. In brief, 25 μg of each plasmid DNA encoding human FLT3L and GM-CSF were mixed into TransIT-QR Hydrodynamic Delivery Solution (Mirus, Madison, WI, USA) for hydrodynamic gene delivery. hNOJ mice were intravenously injected with plasmid solution within 4–6 s using a 27-gauge needle. Seven days after IVT, hNOJ mice were sacrificed after the collection of whole blood.

### Preparation of Primary Cells From hNOJ Mice and Humans

Mouse primary cells were prepared from whole blood, collected by cardiac puncture, and from the spleen of naïve and IVT-hNOJ mice. Human primary cells were prepared from human peripheral blood of healthy Japanese adult volunteers. Mouse splenocytes were prepared from mouse spleens at 7 days post-IVT using the Spleen Dissociation Kit, mouse (Miltenyi Biotec) and the gentleMACS Dissociator (Miltenyi Biotec) according to the manufacturer's instructions. For flow cytometry analysis of individual hNOJ mouse samples, dissociated splenocytes were treated with ACK buffer (0.15 M NH_4_Cl, 1 mM KHCO_3_, and 0.1 mM EDTA-2Na; pH 7.2–7.4) for 1 min at 25°C to lyse the red blood cells, and then suspended in cold DC-staining buffer (PBS containing 2% heat-inactivated fetal bovine serum, 5 mM EDTA-2Na, and 0.01% sodium azide). For isolation of DCs and monocytes using fluorescence-activated cell sorting (FACS), same HSC donor-derived splenocytes were pooled and subjected to EasySep Mouse/human Chimera Isolation kit (StemCell Technologies, Vancouver, BC, Canada) according to the manufacturer's instructions for the enrichment of human leukocytes. Human T and B cells were depleted from the enriched human leukocytes using the CD3 MicroBead (Miltenyi Biotec) and CD19 MicroBead (Miltenyi Biotec) according to the manufacturer's instructions and then suspended in cold sorting buffer (HBSS containing 2% heat-inactivated fetal bovine serum, 5 mM EDTA-2Na, 25 mM HEPES, 100 μg/ml penicillin, and 100 μg/ml streptomycin) until the cell sorting.

PBMCs from hNOJ mice and PBMCs from healthy human donors were separated using Lymphocyte Separation Medium 1077 (PromoCell, Heidelberg, Germany). Naïve CD4^+^ T cells from healthy donors' PBMCs were negatively enriched using the EasySep Human Naïve CD4^+^ T Cell Isolation Kit II (StemCell Technologies) and suspended in IMEM-10 medium [Iscove's Modified Dulbecco's Medium (Thermo Fisher Scientific, Waltham, MA, USA) containing 10% KnockOut Serum Replacement (Thermo Fisher Scientific), 1% GlutaMAX Supplement (Thermo Fisher Scientific), 100 μg/ml penicillin, and 100 μg/ml streptomycin].

### Human Leukocytes Flow Cytometry: Staining, Analysis, and Cell Sorting

The fluorochrome-conjugated monoclonal antibodies (mAbs) used are listed in Table 1. All mAbs were specific for human antigens. Anti-mouse FcγRII/III (2.4G2) mAb (35) and the Human TruStain FcX (BioLegend), which is compatible with flow cytometric staining with anti-human CD16 (3G8) mAb, were used to prevent non-specific binding of mAbs. LIVE/DEAD Fixable Dead Cell Stain Kits (Aqua, Violet, and Near-IR; Thermo Fisher Scientific) were used for staining dead cells, which were gated out during analysis. For flow cytometric analysis, all cells were incubated with blocking antibodies in DC-staining buffer for 20 min on ice. Then, the samples were washed and stained with a mixture of fluorochrome-conjugated mAbs and with LIVE/DEAD Fixable Dead Cell Kit in DC-staining buffer for 30 min on ice. For cell isolation using FACS, the sorting buffer was used instead of DC-staining buffer. For intracellular staining (ICS), after cell surface staining, cells were fixed and permeabilized using eBioscience Foxp3/Transcription Factor Staining Buffer Set (eBioscience/Thermo Fisher Scientific) according to the manufacturer's instructions. Flow cytometry and cell sorting were performed using BD FACSAria III (BD Biosciences, San Diego, CA, USA). Data were saved as FCS files and analyzed using BD FACSDiva 8.0.1 (BD Biosciences) or FlowJo v10.7.1 (Tree Star/BD Biosciences).

**Table 1:** Monoclonal antibodies used for flow cytometry.

**Name**	**Clone**	**Fluorochrome**	**Source**	**Catalog identifier**
CD1c	L161	Alexa Flour 700	BioLegend^f^	Cat# 331530, RRID:AB_2563657
CD3	UCHT1	Brilliant Violet 605 PerCP^a^	BioLegend	Cat# 300460, RRID:AB_2564380 Cat# 300427, RRID:AB_893300
CD4	OKT4	Brilliant Violet 605	BioLegend	Cat# 317438, RRID:AB_11218995
CD5	UCHT2	PE^b^	BioLegend	Cat# 300607, RRID:AB_314093
CD8a	RPA-T8	Alexa Flour 700	BioLegend	Cat# 301027, RRID:AB_493744
CD14	RMO52	FITC^c^	Beckman Coulter^g^	Cat# B36297, RRID:AB_130992
CD16	3G8	PerCP	BioLegend	Cat# 302030, RRID:AB_940380
CD19	HIB19	Brilliant Violet 605	BioLegend	Cat# 302244, RRID:AB_2562015
CD33	P67.6	APC-Cy7^d^	BioLegend	Cat# 366614, RRID:AB_2566416
CD45	HI30	Pacific Blue	BioLegend	Cat# 304029, RRID:AB_2174123
CD56	5.1H11	Brilliant Violet 605	BioLegend	Cat# 362538, RRID:AB_2565856
CD88	S5/1	PE	BioLegend	Cat# 344304, RRID:AB_2067175
CD123	6H6	PE-Cy7^e^	BioLegend	Cat# 306010, RRID:AB_493576
CD141	M80	Brilliant Violet 785	BioLegend	Cat# 344116, RRID:AB_2572195
CD163	GHI/61	PE	BioLegend	Cat# 333605, RRID:AB_1134005
CD301/CLEC10A	H037G3	PE	BioLegend	Cat# 354704, RRID:AB_11219002
CD370/CLEC9A	8F9	APC	BioLegend	Cat# 353806, RRID:AB_2565519
IFN-γ	4S.B3	Brilliant Violet 785	BioLegend	Cat# 502541, RRID:AB_11219192
IL-4	8D4-8	PE-Cy7	Thermo Fisher Scientific	Cat# 25-7049-41, RRID:AB_1659722 Cat# 25-7049-82, RRID:AB_469676
IL-6	MQ2-13A5	PE	BioLegend	Cat# 501106, RRID:AB_315154
IL-17A	eBio64DEC17	APC	Thermo Fisher Scientific	Cat# 17-7179-41, RRID:AB_1582221
HLA-DR	L243	PE	BioLegend	Cat# 307605, RRID:AB_314683
S100A8	REA917	PE	Miltenyi Biotec	Cat# 130-115-353, RRID:AB_2727021
S100A9	MRP 1H9	PE	BioLegend	Cat# 350705, RRID:AB_2564007
TNF-α	MAb11	PE	Thermo Fisher Scientific	Cat# 12-7349-81, RRID:AB_466207
Isotype control				
Mouse IgG1 kappa	MOPC-21	APC Brilliant Violet 785 PerCP PE PE-Cy7	BioLegend	Cat# 400120 Cat# 400170 Cat# 400148 Cat# 400112, RRID:AB_2847829 Cat# 400125, RRID:AB_2861433
Mouse IgG2a kappa	MOPC-173	APC PE	BioLegend	Cat# 400222 Cat# 400212, RRID:AB_326460
Rat IgG1 kappa	RTK2071	PE	BioLegend	Cat# 400407, RRID:AB_326513

a *Peridinin–chlorophyll protein*.

b *Allophycocyanin*.

c *Fluorescein isothiocyanate*.

d *Allophycocyanin-cyanin 7*.

e *Phycoerythrin-cyanin 7*.

f *San Diego, CA, USA*.

g *Brea, CA, USA*.

### Allogeneic Mixed Lymphocyte Reaction

Naïve CD4^+^ T cells prepared from human PBMCs were labeled with 5 μM CellTrace Violet (CTV; Thermo Fisher Scientific) according to the manufacturer's instructions. After labeling, cells were washed twice with the IMEM-10 medium. A total of 2,000–2,500 cells from DC subsets or monocytes of hNOJ mice were FACS-sorted into the U-bottom 96-well plate and were subsequently co-cultured with CTV-labeled allogeneic naïve CD4^+^ T cells at a DC/T cell ratio of 1:20 for 5 days in the IMEM-10 medium at 37°C. On day 5, the CD4^+^ T cells were restimulated with 50 ng/ml phorbol 12-myristate 13-acetate (PMA; Sigma-Aldrich, St. Louis, Mo, USA) and 1 μg/ml ionomycin (Sigma-Aldrich) for 1 h at 37°C. Then, 5 μg/ml Brefeldin A solution was added for 5 h, after which restimulated CD4^+^ T cells were subjected to the flow cytometry as described above.

### Bulk RNA-Sequencing

DC subsets and monocytes from hNOJ mice up to 540 cells isolated using FACS were mixed with 2.7 μl of cold hypotonic lysis buffer consisting of 0.2% (w/v) Triton X-100 (Sigma-Aldrich) and RNase inhibitor (Thermo Fisher Scientific) in 0.2 ml microtubes, immediately frozen on dry ice, and stored at −80°C until cDNA library construction. Cell-lysis solutions were processed to construct cDNA libraries according to the SMART-seq2 protocol (36) with minor modifications. The amplification process was performed with 21 cycles of PCR instead of 18 cycles, and the PCR products were purified using 0.8 × volume of AMPure XP beads (Beckman Coulter). When the amount of PCR products was more than 1 ng, they were used for sequencing library preparation using the Nextera XT DNA library preparation kit (Illumina, San Diego, CA, USA). The libraries were sequenced with 75 bp paired-end reads on an Illumina Miseq (Illumina).

### Bulk RNA-Sequencing Data Processing and Analysis

Adapter sequences and low-quality data were trimmed off from row sequencing reads data of fastq format using flexbar v3.4.0 (37). FastQC v0.11.8 (http://www.bioinformatics.babraham.ac.uk/projects/fastqc) was used to visualize the read quality. Filtered sequencing reads were aligned to the human reference genome (GRCh38.p13 version 32 release; GENCODE) using HISAT2 v2.1.0 (38) with default parameters. The number of reads assigned to genes was calculated using featureCounts v1.6.4 (39).

Differential gene expression between any pair of samples was assessed using R package DESeq2 v1.28.1 (40), with the default false detection rate adjustment of *p*-values for multiple hypothesis testing. For clustering analysis, raw counts were transformed using variance-stabilizing transformation (VST) (41). Hierarchical clustering was performed using Euclidean distance and complete linkage based on all differentially expressed genes (DEGs) among all cell subsets (|Log_2_FC| > 1.5, *p*-value < 0.01) or 488 genes expressed in at least one sample among cell subsets corresponding to Gene Ontology biological functions of “immune system process” (GO: 0002376) annotated using R package biomaRt v2.28.0 (42), and it was displayed on a heatmap generated using R package pheatmap v1.0.12 (43) following Z-score conversion. A volcano plot displaying DEGs between two subsets (CD14^+^CD1c^+^ cells vs. cDC2s, CD14^+^CD1c^+^ cells vs. monocytes) of hNOJ mice was generated using R package EnhancedVolcano v1.6.0 (|Log_2_FC| > 1.5, *p*-value < 0.01) (44).

For hierarchical clustering using gene expression data from RNA-seq data of hNOJ mice and deposited bulk RNA-seq data of humans, raw sequence data for CD5^+^ cDC2 [(30), SRA: SRR10056374, SRR10056375, SRR10056376, and SRR10056377], DC3 [(31), SRA: SRR11832588, SRR11832589, SRR11832590, and SRR11832591], classical monocyte [(45), cMo; SRA: SRR6298336, SRR6298307, SRR6298370, and SRR6298278], intermediate monocyte [(45), iMo; SRA: SRR6298307, SRR6298308, SRR6298371, and SRR6298279], non-classical monocyte [(45), ncMo; SRA: SRR6298338, SRR6298309, SRR6298372, and SRR6298280], Langerhans cell [(46), LC; SRA: SRR7896371, SRR7896374, and SRR7896377], monocyte-derived macrophage [(47), MDM; SRA: SRR8787287, SRR8787291, and SRR8787295], and MoDC [(48), SRA: SRR6815986, SRR6816010, and SRR6815991] were downloaded from SRA (https://trace.ncbi.nlm.nih.gov/Traces/sra/) using parallel-fastq-dump v0.6.6 (https://github.com/rvalieris/parallel-fastq-dump) and processed as described above. In this hierarchical clustering, the 1,000 most variable genes among all the samples were used.

Gene set enrichment analysis (GSEA; https://www.broad.mit.edu/gsea) (49) was used to assess the expression of gene signatures specific for two DC subsets of hNOJ mice. Results were considered significant when normalized enrichment score (NES) was over |1.00| and the *q*-value was below 0.25. GSEA was performed using previously published gene signatures defining human blood cDC2s and DC3s listed in Supplementary Table 2 (29).

### Detection of Inflammatory Responses Using *in vivo* ICS Assay

IVT-hNOJ mice were injected intraperitoneally with 200 μl of PBS containing 2 μg of LPS (O55:B5, Sigma-Aldrich) at 7 days post-IVT. One hour after LPS administration, hNOJ mice were intravenously injected with 500 μl of PBS containing 250 μg of Brefeldin A to measure intracellular cytokine synthesis *in vivo* (50, 51). After 5 h, spleens were collected and immediately dissociated, and the splenocytes were stained to detect intracellular cytokines using flow cytometry as described above.

### Statistical Analyses

Experimental variables were analyzed using the following statistical tests: the unpaired or ratio-paired *t*-test, Mann-Whitney *U* test, normal or repeated-measures one-way ANOVA followed by the Holm-Sidak's multiple comparison test, and two-way ANOVA followed by the Holm-Sidak's multiple comparison test (see individual figure legends). GraphPad Prism software version 6 (GraphPad Software, San Diego, CA, USA) was used for all statistical analyses. A *p*-value < 0.05 was considered statistically significant.

## Results

### CD14^+^CD1c^+^ Cells Are Phenotypically Similar to DC3

**Figure 1:**
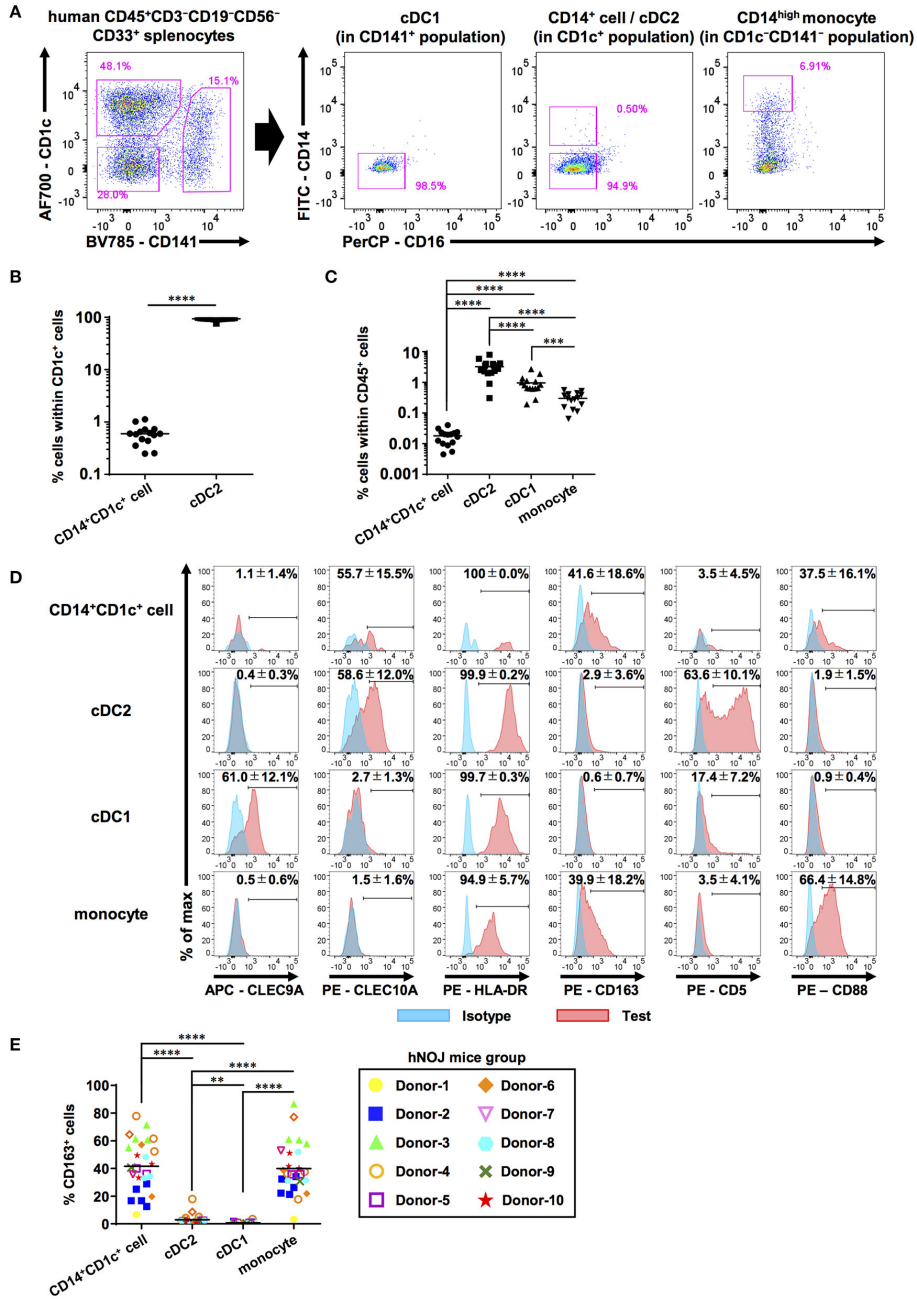
Characterization of human DC populations in hNOJ mice. Cells were prepared from the spleen of humanized NOJ (hNOJ) mice following *in vivo* transfection (IVT). **(A)** A representative gating strategy for cDC1s, cDC2s, CD14^+^CD1c^+^ cells, and CD14^high^ monocytes. **(B)** Individual percentages of CD14^+^CD1c^+^ cells and cDC2s within CD1c^+^ population (*n* = 15). A significant difference (****P < 0.0001) was determined using the ratio-paired *t*-test. **(C)** Individual percentages of CD14^+^CD1c^+^ cells, cDC2s, cDC1s, and monocytes within human CD45^+^ cells (*n* = 15). Significant differences (***P < 0.001, ****P < 0.0001) were determined using the repeated-measures one-way ANOVA followed by the Holm-Sidak's multiple comparison test. **(D)** Representative histogram profiles for subset-associated markers on CD14^+^CD1c^+^ cells, cDC2s, cDC1s, and monocytes using flow cytometry (red: test marker staining, blue: isotype staining). The percentages in each panel show the mean ± SD of marker positive cells in each population (CLEC9A: *n* = 8, CLEC10A: *n* = 4, HLA-DR: *n* = 4, CD163: *n* = 26, CD5: *n* = 5, CD88: *n* = 8). **(E)** Individual percentages of CD163^+^ cells within each cell population related to **(D)**. The same color-symbols show the same donor-derived hNOJ mice. Significant differences (**P < 0.01, ****P < 0.0001) were determined using the repeated-measures one-way ANOVA followed by the Holm-Sidak's multiple comparison test.

Previously, we established a humanized mouse model that enabled the enhanced development of human DC subsets using IVT of human *FLT3L* and *GM-CSF* genes (21). This mouse model was used in this study. The gating strategy for each DC(-like) subset was the same as that in the previous study, except for the following one point (Figure 1A and Supplementary Figures 1A,B). To reduce the number of false-positive cells because of spillover of other fluorochromes, we used an anti-CD14 mAb conjugated with FITC, instead of ECD. We first selected human CD45^+^CD3^−^CD19^−^CD56^−^CD33^+^ splenocytes in hNOJ mice, and then subdivided these cells into three populations: CD141^+^ population (cDC1), CD1c^+^ population (cDC2), and CD1c^−^CD141^−^ (DN) population (Supplementary Figure 1A). CD14 expression in the three populations was comparatively plotted based on the gating threshold that was set by the isotype control staining (Figure 1A and Supplementary Figure 1B). cDC1 population was negative for CD14 expression, and DN population expressed CD14 at various levels (Figure 1A). For human PBMCs, the DN population harbored classical monocyte populations that exhibited CD14^high^CD16^−^ phenotype (Supplementary Figure 1C). When CD14^+^ cells in DN populations of hNOJ mice were divided into CD14^high^ cells and CD14^low^ cells, CD14^high^ DN cells highly expressed CD88, a recently defined marker for the monocyte (30, 31), however, CD14^low^ DN cells exhibited heterogeneous CD88 expression (Supplementary Figure 1D). Taken together, we gated a CD14^high^CD16^−^ fraction in DN population of hNOJ mice as a putative classical monocyte subset. In addition to these expected results, we noticed the presence of a small CD14^+^ fraction within the cDC2 population (~1% within the CD1c^+^ population) of hNOJ mice (Figure 1A). CD14^+^ cells were detectable in all humanized mice (*n* = 15), but the frequencies were 150-fold less than those of CD14^−^CD1c^+^ cDC2 cells (Figures 1B,C). When human PBMCs were analyzed by the same gating strategy, CD14^+^ cells were also found in CD1c^+^ population with CD14^high^ and CD14^low^ population (Supplementary Figure 2A). CD14^low^CD1c^+^ cells and CD14^−^CD1c^+^ cells (cDC2s) in human PBMCs were negative for CD88 (Supplementary Figure 2B). Moreover, these CD14^low^CD1c^+^ cells highly expressed CD163 (Supplementary Figure 2C), suggesting that our gating strategy indeed identifies human DC3 subset as CD14^low^CD1c^+^ cells. Then, we compared the ratios of CD14^+^CD1c^+^ cells vs. cDC2s between splenocytes in hNOJ mice and human PBMCs, and found that they were approximately seven times higher in human PBMCs than in splenocytes of hNOJ mice (Supplementary Figure 2D). Nevertheless, this atypical cellular subset, as well as cDC1s and cDC2s, was found at increased frequencies in IVT-hNOJ mice compared to untreated hNOJ mice (Supplementary Figure 2E). Thus, we identified CD14^+^CD1c^+^ cells that were induced in IVT-hNOJ mice at elevated frequencies.

Next, we examined the expression of other DC- and monocyte/macrophage-related surface markers (CLEC9A, CLEC10A, HLA-DR, CD163, CD5, and CD88) on these subsets in the spleens of hNOJ mice (Figure 1D). CLEC9A and CLEC10A were selectively expressed in cDC1 and cDC2, respectively, as expected since they have been previously utilized as cDC1 and cDC2 markers (52, 53). Interestingly, CD14^+^CD1c^+^ cells expressed cDC2 marker CLEC10A at frequencies similar to cDC2s, but CD14^+^ monocyte-like cells did not. We verified the expression of HLA-DR in all four subsets in hNOJ mice, in corroboration with the results of the previous study (54), and HLA-DR level on CD14^+^CD1c^+^ cells was closer to that observed on cDC2s than to the HLA-DR level observed on monocytes. Thus, our CLEC10A and HLA-DR expression data support a similarity between CD14^+^CD1c^+^ cells and cDC2s.

Recently, a new DC subset was identified that is phenotypically different from cDC1 and cDC2; therefore, it was denominated DC3 (29). To further characterize CD14^+^CD1c^+^ cells, we examined the surface expression of other molecules that are differentially expressed among cDC1, cDC2, and DC3. CD163, a phenotypic marker for DC3 (29, 30, 55), was selectively expressed on CD14^+^CD1c^+^ cells, although the frequency of CD163^+^ cells varied depending on the HSC donor (Figure 1E). In contrast, the cDC2-related marker, CD5 (56), was selectively expressed on cDC2, but not on CD14^+^CD1c^+^ cells. The monocyte marker, CD88, was highly expressed on monocytes of hNOJ mice. In CD14^+^CD1c^+^ cells, some cells expressed CD88, but its expression was lower than monocytes like the CD14 expression pattern. Taken together, these results indicate that the phenotype of CD14^+^CD1c^+^ cells from hNOJ mice is highly similar to human DC3 phenotype.

### CD14^+^CD1c^+^ Cells Are Functionally Competent for Priming and Polarizing Naïve CD4^+^ T Cells

**Figure 2:**
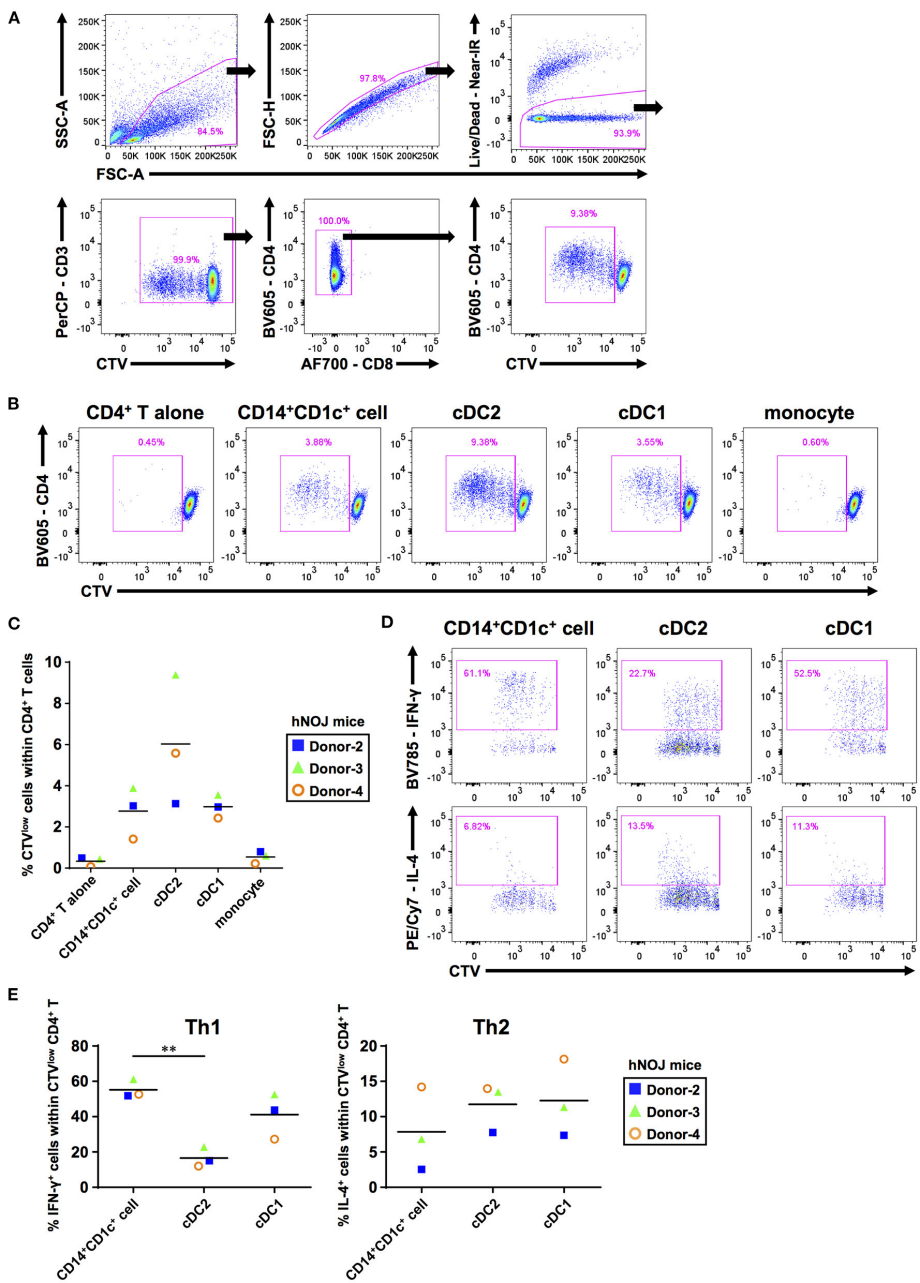
The ability to prime and polarize Th cells among human dendritic cell populations derived from hNOJ mice. DCs and monocytes were isolated from pooled spleens of donor-matched hNOJ mice following IVT, and CD4^+^ T cells were prepared from allogeneic human peripheral blood mononuclear cells. CD4^+^ T cells were subjected to flow cytometric analysis following co-culture for 5 days with DCs or monocytes. **(A)** A representative gating strategy for CD4^+^ T-cell proliferation. **(B)** Representative flow cytometry profiles of CD4^+^ T-cell proliferation based on the CellTrace Violet (CTV) intensity in each co-culture condition. **(C)** Individual percentages of CTV^low^ cells within CD4^+^ T cells. The same color symbols show the same donor-derived hNOJ mice. The repeated-measures one-way ANOVA followed by the Holm-Sidak's multiple comparison test was used to compare among co-culture conditions, and no significant differences were observed. **(D)** Representative flow cytometry profiles of IFN-γ* and* IL-4 expression in CD4^+^ T cells. **(E)** Individual percentages of IFN-γ^+^ cells (Th1; *n* = 3) and IL-4^+^ cells (Th2; *n* = 3) within CTV^low^CD4^+^ T cells. The same color symbols show the same donor-derived hNOJ mice. Significant differences (**P < 0.01) were determined using the repeated-measures one-way ANOVA followed by the Holm-Sidak's multiple comparison test.

We addressed whether CD14^+^CD1c^+^ cells can activate naïve CD4^+^ T cells. First, we performed an allogeneic mixed lymphocyte reaction to evaluate CD4^+^ T-cell priming capabilities of these populations. CD14^+^CD1c^+^ cells, cDCs, and monocytes were isolated and co-cultured with CTV-labeled allogeneic naïve CD4^+^ T cells derived from human PBMCs. After co-culture for 5 days, the proliferation of CD4^+^ T cells was monitored by CTV degradation using flow cytometry (Figures 2A,B). Co-cultures of CD14^+^CD1c^+^ cells, cDC2s, and cDC1s induced proliferation of naïve CD4^+^ T cells, whereas co-culture of monocytes induced little proliferation of naïve CD4^+^ T cells at the level similar to the culture condition of CD4^+^ T cells alone (Figure 2C).

After T-cell priming, DCs polarize naïve CD4^+^ T cells into divergent T-cell subsets depending on DC functions (57). Therefore, it is important to clarify which types of T cell subsets are induced following interaction with individual DC subsets. After co-culture for 5 days with either CD14^+^CD1c^+^ cells, cDC2s, or cDC1s, CD4^+^ T cells were restimulated with PMA and ionomycin for 6 h. Then, we evaluated the frequencies of proliferated CD4^+^ T cells (CTV^low^CD4^+^ T cells) that produced Th1 and Th2 cytokines (IFN-γ and IL-4, respectively) using ICS (Figure 2D, Supplementary Figure 3A). We first examined the frequency of these CD4^+^ T cells within total CD4^+^ T cells to evaluate how much the co-cultured DC subset polarized naive CD4^+^ T cells. However, since the degree of cell proliferation among samples varied greatly, there is no significant difference in the amount of polarized CD4^+^ T cells (Supplementary Figure 3B). Therefore, to accurately characterize the induced polarization, we focused only on proliferated CD4^+^ T cells and compared the frequency of cytokine-producing cells, and observed that CD14^+^CD1c^+^ cells have greater Th1-polarizing capacity compared with cDC2 (Figure 2E). These results indicate that CD14^+^CD1c^+^ cells can be discriminated from cDC2s by their functional aspects. Of note, a previous study has shown similar Th1-polarizing capacity in DC3s of human PBMCs (31). Thus, the Th1-polarizing capacity, in addition to phenotypic markers, highlights the similarity between CD14^+^CD1c^+^ cells and DC3s. We also evaluated Th17-polarizing capacity based on IL-17A expression, another characteristic of DC3s (30). However, we did not observe IL-17A^+^CD4^+^ T cells within CD4^+^ T cells that were stimulated with CD14^+^CD1c^+^ cells, cDC1s, and cDC2s (Supplementary Figures 3C,D). Collectively, our data demonstrate a functional similarity between CD14^+^CD1c^+^ cells from hNOJ mice and DC3s from human PBMCs.

### Transcriptional Profile Reveals DC3-Specific and Inflammatory Signatures in CD14^+^CD1c^+^ Cells

**Figure 3:**
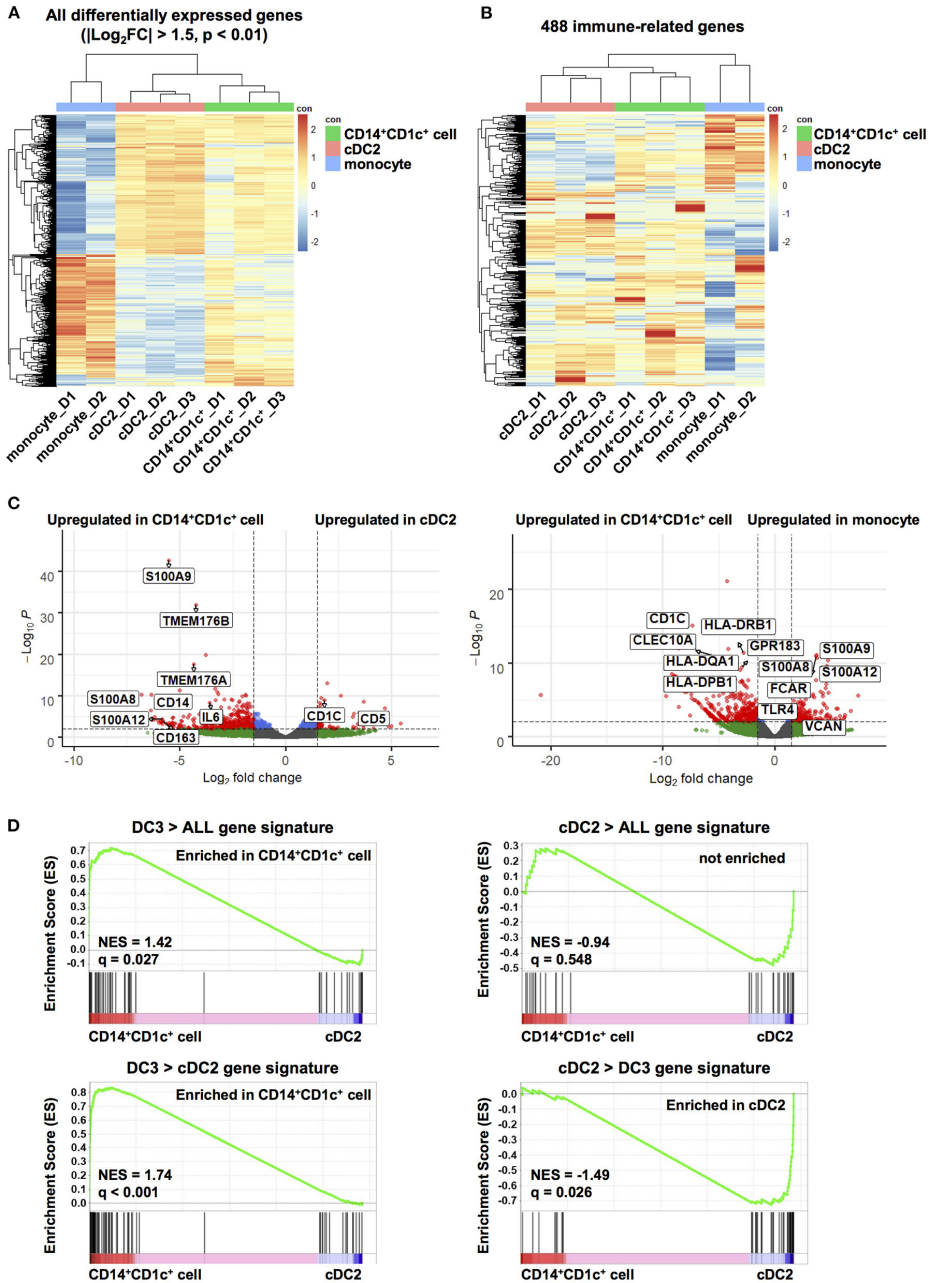
Transcriptome analysis of human dendritic cell populations in hNOJ mice. Total RNA was extracted from CD14^+^CD1c^+^ cells, cDC2s, and monocytes isolated from the spleen of IVT-hNOJ mice and sequenced. **(A)** Heatmap visualization of the z-scores for the all DEGs among CD14^+^CD1c^+^ cells (*n* = 3), cDC2s (*n* = 3), and monocytes (*n* = 2) obtained (|Log_2_FC| > 1.5, *p*-value < 0.01) using the hierarchical clustering analysis. **(B)** Heatmap visualization of the z-scores for 488 genes expressed in at least one sample among CD14^+^CD1c^+^ cells (*n* = 3), cDC2s (*n* = 3), and monocytes (*n* = 2) obtained using the hierarchical clustering analysis using with the sets of genes corresponding to Gene Ontology annotation “immune system process.” The colors in the heatmap indicate high (red) or low (blue) expression across the sample set. **(C)** Volcano plots displaying DEGs between two subsets in hNOJ mice (CD14^+^CD1c^+^ cells vs. cDC2s, CD14^+^CD1c^+^ cells vs. monocytes). Genes with |Log_2_(fold change)| > 1.5, and *p*-value < 0.01 were considered significant (red plot). **(D)** GSEA comparing CD14^+^CD1c^+^ cells and cDC2s derived from hNOJ mice using published cDC2s and DC3s gene signatures (29). Statistical significance was defined by normalized enrichment score (NES) and *q*-value calculated with GSEA software using the default parameter.

We characterized transcriptional profiles of CD14^+^CD1c^+^ cells and compared them with those of cDC2s and monocytes. CD14^+^CD1c^+^ cells, cDC2s, and monocytes were isolated from splenocytes pooled from same donor HSC-derived hNOJ mice. Three mouse groups from different donors were used. When RNA-seq analysis was carried out, one analysis from monocytes was excluded because cDNA amplification was insufficient. First, to elucidate whether CD14^+^CD1c^+^ cells were transcriptionally profiled as a population closer to the cDC2 population or to the monocyte population, we performed the hierarchical clustering using all DEGs among all cell subsets (Figure 3A; |Log_2_FC| > 1.5, *p*-value < 0.01). Three clusters of each cell subset were formed, and the cluster of CD14^+^CD1c^+^ cells was found to be closer to cDC2s than to monocytes. We also performed the hierarchical clustering of 488 genes, which are annotated in Gene Ontology database as contributing to “immune system process” and expressed in at least one sample (Figure 3B). Similar to the result of clustering using DEGs, the cluster of CD14^+^CD1c^+^ cells was closer to cDC2s than monocytes. Interestingly, both groups of genes upregulated in cDC2 clusters and monocyte clusters tended to be upregulated in clusters of CD14^+^CD1c^+^ cells (Figure 3A), suggesting that CD14^+^CD1c^+^ cells have both transcriptional characteristics of cDC2 and monocyte. Therefore, we compared the similarity of CD14^+^CD1c^+^ cells of hNOJ mice to the human CD1c^+^ DC subsets or monocyte-related subset using public data. The results showed that CD14^+^CD1c^+^ cells were closest to cDC2 of hNOJ mice, followed by human-derived cDC2 and DC3 clusters (Supplementary Figure 4).

To further characterize CD14^+^CD1c^+^ cells, we identified DEGs between CD14^+^CD1c^+^ cells vs. cDC2s and CD14^+^CD1c^+^ cells vs. monocytes in hNOJ mice (|Log_2_FC| > 1.5, *p*-value < 0.01). All DEGs were listed in Supplementary Table 3, and genes in this table that have been previously reported to be characteristic of human DC3, cDC2, and monocyte (29, 31, 32) were selected and labeled in the volcano plot (Figure 3C). In a comparison of CD14^+^CD1c^+^ cells and cDC2 of hNOJ mice, CD14^+^CD1c^+^ cells highly expressed *CD14* and *CD163*, whereas cDC2s expressed *CD1c* and *CD5*. These transcriptional data were consistent with the surface expression profiles shown in Figure 1. In addition, we observed the upregulation of *S100A8, S100A9*, and *IL-6* in CD14^+^CD1c^+^ cells, which are related to inflammation (58). Moreover, CD14^+^CD1c^+^ cells exhibited higher expression of DC3-related genes, including *S100A12, TMEM176A*, and *TMEM176B* (29, 31). In a comparison of CD14^+^CD1c^+^ cells and monocytes of hNOJ mice, similar to previous reports of DEGs in human DC3s and monocytes (31, 32), CD14^+^CD1c^+^ cells highly expressed *CD1c, CLEC10A, GPR183, HLA-DQA1* and *HLA-DPB1*, while monocytes highly expressed *S100A8, S100A9, S100A12, FCAR, TLR4* and *VCAN*.

GSEA clarifies any enrichment of specific gene signatures in a pairwise comparison of gene expression data derived from two cell populations (49); therefore, it was applied to determine the similarity between CD14^+^CD1c^+^ cells from hNOJ mice and DC3s from human PBMCs. We used published gene signatures of DC3s (DC3 > ALL and DC3 > cDC2) and cDC2s (cDC2 > ALL and cDC2 > DC3) (Supplementary Table 2), according to Villani et al. where DC3/cDC2 was compared with other DC subsets in which CD14^+^ cells were excluded (29). DC3 gene signatures (DC3 > ALL and DC3 > cDC2) were significantly enriched in CD14^+^CD1c^+^ cells (Figure 3D; DC3 > ALL: NES = 1.42, *q* = 0.027; DC3 > DC2: NES = 1.74, *q* < 0.001), whereas a cDC2 gene signatures (cDC2 > DC3) were significantly enriched in cDC2s from hNOJ mice (Figure 3D; NES = −1.49, *q* = 0.026). Therefore, these results indicate that CD14^+^CD1c^+^ cells from hNOJ mice are a subset closely related to DC3s from human PBMCs, based on transcriptional profiles.

### CD14^+^CD1c^+^ Cells Express Pro-inflammatory Mediators by LPS Administration

Since transcriptional analysis revealed the inflammatory signatures of CD14^+^CD1c^+^ cells, we next examined whether CD14^+^CD1c^+^ cells exert a pro-inflammatory response in acute inflammation.

**Figure 4:**
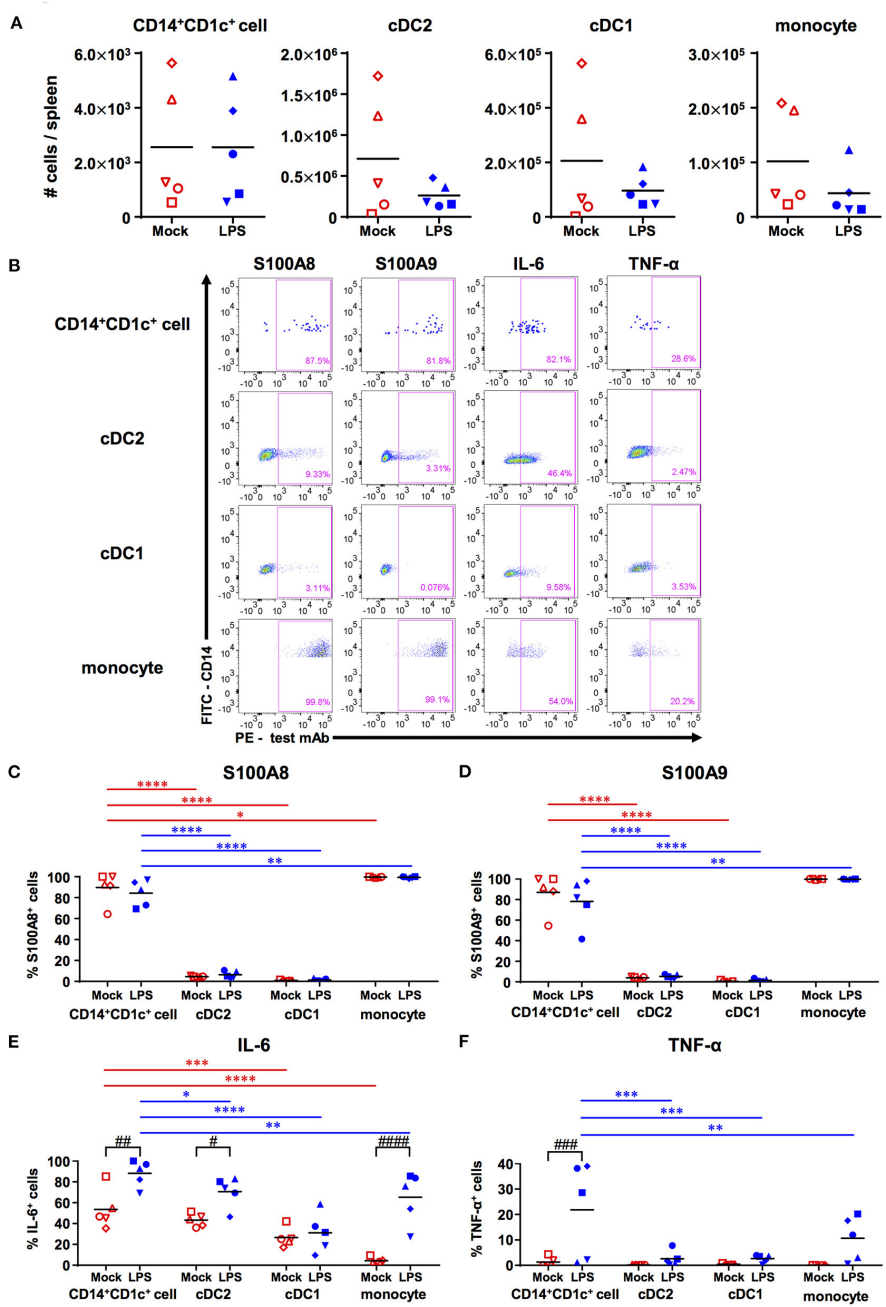
Responsiveness of human dendritic cell populations in hNOJ mice against LPS-induced acute inflammation. Cells were prepared from the spleen of IVT-hNOJ mice following intraperitoneal LPS injection. **(A)** Individual absolute cell numbers of CD14^+^CD1c^+^ cells, cDC2s, cDC1s, and monocytes within human CD45^+^ cells (Mock: *n* = 5, LPS: *n* = 5). The Mann-Whitney *U* test was used, and no significant differences were observed. The distinct symbols show each separate hNOJ mouse. **(B)** Representative flow cytometry profiles of intracellular inflammation-related proteins/cytokines (S100A8, S100A9, IL-6, and TNF-α) in cell populations. **(C–F)** Individual percentages of the cells expressing **(C)** S100A8, **(D)** S100A9, **(E)** IL-6, and **(F)** TNF-α (Mock: *n* = 5, LPS: *n* = 5). Significant differences (*P < 0.05, **P < 0.01, ***P < 0.001, ****P < 0.0001) between CD14^+^CD1c^+^ cells and each other subset under the same conditions (red: mock, blue: LPS) were determined using the repeated-measures two-way ANOVA followed by the Holm-Sidak's multiple comparison test. In addition, significant differences (^#^*P* < 0.05, ^##^*P* < 0.01, ^###^*P* < 0.001, and ^####^*P* < 0.0001) between Mock and LPS conditions in same cell subsets were determined by repeated-measures two-way ANOVA followed by the Holm-Sidak's multiple comparison test. The distinct symbols show each separate hNOJ mouse.

When LPS was administrated to hNOJ mice to produce acute inflammation, we observed that LPS had little effect on the absolute numbers of all myeloid cell subsets (CD14^+^CD1c^+^ cells, cDC1s, cDC2s, and monocytes), although there was a large variation among individual mice (Figure 4A).

Then, we analyzed the expression of two intracellular inflammation-related proteins, namely S100A8 and S100A9 (also known together as “calprotectin”), and of two cytokines, namely IL-6 and TNF-α, in each cell subset using flow cytometry and compared their levels between steady (non-LPS-stimulated) state and acute inflammatory state (Figure 4B). The gating threshold of S100A8^+^ and S100A9^+^ cells was set with reference to the flow cytometry profiles of cDC2, which does not express S100A8 and S100A9 (31). The gating threshold of IL-6^+^ and TNF-α^+^ cells was set by the isotype control staining (Supplementary Figures 5A,B). As a result, we found that most CD14^+^CD1c^+^ cells and monocytes, but not cDC1s and cDC2s, expressed S100A8 and S100A9, regardless of LPS administration (Figures 4C,D). In addition, the frequencies of IL-6 producing cells were significantly higher in CD14^+^CD1c^+^ cells than in all subsets except for cDC2s in steady state (Figure 4E). After LPS injection, the frequencies of IL-6 producing cells were elevated in all subsets except for cDC1s, but CD14^+^CD1c^+^ cells reached the highest numbers of IL-6-producing cells among all subsets from the same mice (Figure 4E). Similar to DC3s (31), we also found TNF-α expression in CD14^+^CD1c^+^ cells after LPS injection (Figure 4F). Collectively, our data show that CD14^+^CD1c^+^ cells constitutively produce inflammatory-related calprotectin and upregulate pro-inflammatory cytokines under acute inflammatory conditions in humanized mice.

## Discussion

Since DC3s have been proposed as a DC subpopulation distinct from cDC2s (29), the functional and transcriptional profiles of DC3s have been investigated in several studies using human peripheral blood (30–32). However, tissue-resident DC3s remain uncharacterized owing to limited access to tissue cells. Therefore, it is desirable to establish a small animal model that is able to reproduce the development of DC3s. Here, we have identified inflammatory CD14^+^CD1c^+^ DCs in humanized mice as the equivalent to DC3s in humans.

In this study, we investigated whether humanized mice are able to develop a DC subpopulation similar to human DC3s by focusing on the identity of CD14^+^ cells within the CD1c^+^ cell population. To examine this, we used our previously established humanized mouse model in which human FLT3L and GM-CSF were expressed by using an IVT strategy (21). In our previous study (21), expression of human FLT3L and GM-CSF enhanced cDC1 and cDC2 development. This study shows that it also promoted CD14^+^CD1c^+^ cell development (Supplementary Figure 2E). Recent reports regarding the ontogeny of DC3s indicate that enhanced differentiation of DC3s can be achieved through administration of FLT3L and/or GM-CSF (30, 31). Therefore, we considered our FLT3L- and GM-CSF-IVT strategy reasonable to investigate the development of DC3s in humanized mice. However, in this study, we encountered difficulties in analyzing all DC subsets in the peripheral blood of hNOJ mice because of low yield, notwithstanding FLT3L and GM-CSF expression. Because of this limitation, we were unable to compare the CD14^+^CD1c^+^ cells in the spleen with those in the blood. It will be necessary to clarify whether the DC3-like CD14^+^CD1c^+^ cells observed in the spleen are differentiated *in situ* from the progenitor or have entered the spleen from the blood.

The CD1c^+^ population from hNOJ mice consisted of heterogeneous DCs with differential expression of CD5, CD14, and CD163 (Figure 1D), similar to the CD1c^+^ population from human peripheral blood (29, 30, 56). In general, during flow cytometric analysis, CD14^+^ cells are first gated out, in order to separate cDCs from CD14^+^ monocytes. In contrast, we first focused on the DC-like population with cDC-specific markers, followed by fractionation of DCs depending on the CD14 expression level (21). This gating strategy allowed us to capture DC3-like CD14^+^CD1c^+^ cells from a heterogeneous CD1c^+^ population (Figures 1A,B). Recent studies analyzing DC3s isolated from human PBMCs also adopted similar gating strategies, in which CD14-positive cells were not excluded (30, 31). Although these studies used a different set of markers to identify DC3s (e.g., CD5, CD163, BTLA), they consistently used CD14 marker as one of the selecting markers for DC3s. These studies showed much larger DC3 populations (DC3:cDC2 = 1:2 to 1:6) in human PBMCs (30, 31) than our CD14^+^CD1c^+^ cell population (CD14^+^CD1c^+^ cell:cDC2 = 1:150) in the spleen of hNOJ mice (Figure 1B). When we analyzed human PBMCs using the same gating strategy as the analysis for hNOJ mice (Supplementary Figure 2A), the ratio of CD14^low^CD1c^+^ cells to cDC2s was 1:20 and was lower than the previous studies (30, 31), possibly due to different gating strategies. However, the ratio was still higher than that of hNOJ mice (Supplementary Figure 2D). This difference in the ratio of DC3 (CD14^+^CD1c^+^ cell population) to cDC2 between human and hNOJ mice may be due to the difference in developmental conditions within humanized mouse models and humans, including the lack of all human cytokines other than FLT3L and GM-CSF in hNOJ mice. CD14^+^CD1c^+^ cells found in humanized mice may not fully represent *bona fide* DC3s because of different human cytokine milieu and low expression of CD88 in some CD14^+^CD1c^+^ cells. However, our identification of a DC subset distinct from cDC2s, on the basis of CD14 expression level, may be an important finding toward unifying the fractionation of DC3s, which still varies among research groups.

In human myeloid cells, CD14 and CD1c double-positive cells include not only DC3s but also monocyte-derived macrophages and MoDCs (30, 59–61). Unlike macrophages, DCs are known to be fully capable of activating naïve T cells (61, 62). We showed that CD14^+^CD1c^+^ cells as well as other cDCs, primed and promoted the proliferation of naïve CD4^+^ T cells at higher levels than did monocytes (Figure 2C), supporting a distinct functionality of CD14^+^CD1c^+^ cells from macrophages. Moreover, it is difficult to distinguish between DC3s and MoDCs because their functionality and transcription profiles tend to overlap conspicuously and no discriminative markers have been reported yet (13). However, our transcriptional analysis demonstrated that CD14^+^CD1c^+^ cells isolated from hNOJ mice are closer to cDC2s and DC3s than to MoDCs in humans (Supplementary Figure 4). In addition, the development of inflammatory MoDC is believed to require IL-4 (63, 64), and the development of CD14^+^CD1c^+^ cells in hNOJ mice was enhanced in the absence of human IL-4 (Supplementary Figure 2E). These results indicated that CD14^+^CD1c^+^ cells isolated from hNOJ mice could be discriminated from monocyte-derived macrophages and MoDCs.

Recent studies on DC3s share a common understanding that CD14^+^ DC3s, as well as cDC2s, stimulate and induce proliferation of naïve T cells (29–31). Bourdely et al. (31) showed that naïve T cells could be polarized into Th1 cells, whereas Dutertre et al. (30) showed that they could be significantly polarized into Th17 cells but not into Th1 cells. This difference may depend on the activation status of DCs: the former study used DC3s that was activated using multiple TLR ligands after isolation (31), and the latter study used unstimulated DC3s after isolation (30). In the present study, we demonstrated that CD14^+^CD1c^+^ cells polarized Th1 cells but not Th17 cells (Figure 2E and Supplementary Figure 3D), corroborating with the results of the study by Bourdely et al. (31) regarding the properties of DC3s. Since we previously observed that IVT of hNOJ mice with FLT3L- and GM-CSF-encoding plasmids could enhance the activation/maturation of cDCs (21), it is likely that activated CD14^+^CD1c^+^ cells may show similar properties to DC3s as reported by Bourdely et al. (31).

Although CD14^+^CD1c^+^ cells did not polarize Th17 cells, CD14^+^CD1c^+^ cells markedly produced pro-inflammatory cytokines IL-6 and TNF-α in LPS-induced acute inflammation (Figures 4E,F). These pro-inflammatory signatures were similar to DC3s, which have been reported to be more potent producers of pro-inflammatory cytokines (namely IL-1β, IL-8, and TNF-α) than cDC2s under various stimulated conditions (30–32). In addition, most of CD14^+^CD1c^+^ cells constitutively produced S100A8 and S100A9, regardless of the LPS administration (Figures 4C,D). S100A8 and S100A9 are collectively known as calprotectin, mainly produced by neutrophils and monocytes/macrophages in response to inflammation by autoimmunity or infection (58, 65). Moreover, recent studies using RNA-seq analysis have shown that mRNA of S100A8 and S100A9 is constitutively expressed in DC3s derived from healthy donors (29–31). These results suggest that CD14^+^CD1c^+^ cells are a potent pro-inflammatory DC subset, like DC3s.

The gene signature adopted for DC3s in the transcriptional analysis in recent studies (30, 31), including our present one, is based on the results of Villani et al. (29). Our bulk RNA-seq performed on CD14^+^CD1c^+^ cells showed a gene profile close to *bona fide* DC3s (Figure 3D), because gene signatures of DC3s (DC3 > cDC2 and DC3 > ALL) reported by Villani et al. (29) were enriched in CD14^+^CD1c^+^ cells. However, the gene signature of cDC2s (cDC2 > ALL) (29) overlapped in CD14^+^CD1c^+^ cells and could not clearly distinguish between CD14^+^CD1c^+^ cells and cDC2s of hNOJ mice (Figure 3D). In addition, although Cytlak et al. reported that DC3s expressed higher levels of IL-1β than cDC2 (32), our RNA-Seq results showed that the expression levels of IL-1β transcripts were comparable between CD14^+^CD1c^+^ cells and cDC2, resulting in unidentified *IL-1*β in DEGs (Supplementary Table 3). The discrepancy would be due to the different cell isolation/separation strategy, e.g., we isolated CD14^+^ cells from CD1c^+^ cells as a DC3 counterpart, whereas Villani et al. (29) separated between DC3s and cDC2s among CD14^−^CD1c^+^ cells by scRNA-seq analysis. Indeed, CD14^+^CD1c^+^ cells in hNOJ mice exhibited heterogeneous surface CD163 expression (Figure 1E), whereas *bona fide* DC3s and cDC2s in human PBMCs were clearly distinguished as CD163-positive cells and CD163-negative cells, respectively (29). In the future, it will be necessary to clarify which subpopulations among CD14^+^CD1c^+^ cells are equivalent to *bona fide* DC3s, based on heterogeneous CD163 expression utilizing single-cell RNA-seq.

In conclusion, our phenotypical, transcriptional, and functional analyses showed that CD14^+^CD1c^+^ cells were distinct DC subsets from cDC2s even in the same CD1c^+^ population, and that the characteristics of CD14^+^CD1c^+^ cells were similar to those of recently described DC3s (29–31). Therefore, our results provide further proof for the utilization of the humanized mouse model, which enables the reconstruction of human DC heterogeneity as cDC2s and DC3s within the CD1c^+^ population. Given the current lack of DC3 counterparts in mice and the recent reports of DC3-specific progenitor cells (31, 32), our humanized mouse model is expected to provide a useful platform to clarify *in vivo* ontogeny and dynamics of DC3s. Additionally, this humanized mouse model may also be helpful to investigate the response of DC3s to specific pathogens in future studies.

## Data Availability Statement

We have deposited RNA-seq data in Sequence Read Archive (https://www.ncbi.nlm.nih.gov/sra). Accession number is PRJNA687607.

## Ethics Statement

Human umbilical cord blood samples were obtained from donors after receiving written informed consent, and they were donated by the Japanese Red Cross Society Kanto-Koshinetsu Block Blood Center (Tokyo, Japan). Human peripheral blood samples were obtained from healthy Japanese adult volunteers, after receiving written informed consent. The use of human cord blood and peripheral blood was approved by the Medical Research Ethics Committee of the NIID for the Use of Human Subjects (protocol numbers 835 and 887, respectively). All mice were treated in accordance with the guidelines of the Institutional Animal Care and Use Committee of the NIID (protocol numbers 117027, 118056, and 119024).

## Author Contributions

RIwab, KT, and YT: study design. RIwab, KI, KT, and YT: data curation. RIwab, KT, RW, and RIwak: acquisition of data. RIwab, KI, KT, RW, YT-Y, and YT: analysis and interpretation of data. RIwab, KI, KT, RW, HM, YT-Y, HT, and YT: validation. RIwab and KI: writing the original manuscript. KT and YT: review and/or revision of the manuscript. All authors: contributed to the article and approved the submitted version.

## Conflict of Interest

The authors declare that the research was conducted in the absence of any commercial or financial relationships that could be construed as a potential conflict of interest.

## Supplementary Material

Supplementary Table 1 List of hNOJ mice used in the present study.

Supplementary Table 2 Gene signatures used in GSEA analysis.

Supplementary Table 3 List of differentially expressed genes in the present study.

Supplementary Figure 1 Flow cytometric gate setting of human DC and monocyte populations in the present study. Cells were prepared from the spleen of hNOJ mice following IVT and human peripheral blood. **(A)** A representative gating strategy for CD141^+^ population, CD1c^+^ population, and CD1c^−^CD141^−^ (DN) population of hNOJ mice. **(B)** Representative flow cytometry profiles for cDC1s, cDC2s, CD14^+^CD1c^+^ cells, and CD14^high^ monocytes with anti-CD14 monoclonal antibody (mAb) staining (upper panels) and with its isotype control staining (lower panels). **(C)** A representative flow cytometry profile for CD14^high^CD16^−^ classical monocytes in DN population of human PBMCs under the same staining condition of hNOJ mice samples. **(D)** Representative flow cytometry profiles for CD14^high^ and CD14^low^ cells in DN population of hNOJ mice. The histogram shows the PE-fluorescence intensity of CD14^high^ cells (red: anti-CD88 mAb staining, orange: isotype control staining) and CD14^low^ cells (blue: anti-CD88 mAb staining, green: isotype control staining).

Supplementary Figure 2 Characterization of human DC and monocyte populations in humans and hNOJ mice. Cells were prepared from human peripheral blood and the spleen of naïve hNOJ mice or hNOJ mice following IVT. **(A)** Representative flow cytometry profiles for CD1c^+^ population and DN population of human PBMCs. **(B)** A representative histogram profile of CD88 expression on CD1c^+^ population and DN population of human PBMCs [red: CD14^low^CD1c^+^ cells, orange: CD14^−^CD1c^+^ cells (cDC2), blue: CD14^high^CD1c^+^ cells, CD14^high^CD16^−^ DN cells (classical monocyte)]. **(C)** Representative histogram profiles of CD163 expression on CD14^low^CD1c^+^ cells, cDC2s, and classical monocytes in human PBMCs (red: test marker staining, blue: isotype control staining). The percentages in each panel show the mean ± SD of marker positive cells in each population (*n* = 5). **(D)** Individual percentages of CD14^+^(CD14^low^)CD1c^+^ cells and cDC2s within CD1c^+^ population in hNOJ mice (*n* = 15) and humans (*n* = 5). A significant difference (^***^*P* < 0.001) was determined using the Mann-Whitney *U* test. **(E)** Individual percentages of CD14^+^CD1c^+^ cells, cDC2s, cDC1s, and monocytes within human CD45^+^ cells in naïve hNOJ mice (*n* = 5) and IVT-hNOJ mice (*n* = 15). Significant differences (^**^*P* < 0.01, ^***^*P* < 0.001) were determined using the Mann-Whitney *U* test.

Supplementary Figure 3 Expression of IFN-γ, IL-4, and IL-17A in CD4^+^ T cells co-cultured with human DC subsets. **(A)** Gating strategy for IFN-γ^+^ cells (upper panels) and IL-4^+^ cells (lower panels) using identical cDC2 co-cultured CD4^+^ T cell samples stained with test and isotype antibodies. **(B)** Individual percentages of IFN-γ^+^CTV^low^ cells (Th1; *n* = 3) and IL-4^+^CTV^low^ cells (Th2; *n* = 3) within total CD4^+^ T cells. The Same color symbols show the same donor-derived hNOJ mice. The repeated-measures one-way ANOVA followed by the Holm-Sidak's multiple comparison test was used, and no significant differences were observed. **(C)** Representative flow cytometry profiles of IL-17A^+^ cells within CD4^+^ T cells. **(D)** Individual percentages of IL-17A^+^CTV^low^ cells within total CD4^+^ T cells and IL-17A^+^ cells within CTV^low^CD4^+^ T cells. The same color symbols show the same donor-derived hNOJ mice. The repeated-measures one-way ANOVA followed by the Holm-Sidak's multiple comparison test was used, and no significant differences were observed.

Supplementary Figure 4 Hierarchical clustering analysis among CD1c^+^ DC subsets and monocyte-related subsets in hNOJ mice and humans. Heatmap visualization of the z-scores for the 1,000 most variable genes among hNOJ mice samples [CD14^+^CD1c^+^ cell (*n* = 3), cDC2 (*n* = 3), and monocyte (*n* = 2)] and human samples [CD5^+^ cDC2 (*n* = 4), DC3 (*n* = 4), classical monocyte (cMo; *n* = 4), intermediate monocyte (iMo; *n* = 4), non-classical monocyte (ncMo; *n* = 4), monocyte-derived DC (MoDC; *n* = 3), monocyte-derived macrophage (MDM; *n* = 3), and Langerhans cell (LC, *n* = 3)] using the hierarchical clustering analysis.

Supplementary Figure 5 Flow cytometric gate setting of IL-6^+^ cells and TNF-α^+^ cells in cell subsets. Cells were prepared from the spleen of IVT-hNOJ mice following intraperitoneal LPS injection. **(A)** Representative flow cytometry profiles for CD14^+^CD1c^+^ cells, cDC2s, cDC1s, and monocytes with anti-IL-6 mAb staining (left panels) and with its isotype control staining (right panels). **(B)** Representative flow cytometry profiles for CD14^+^CD1c^+^ cells, cDC2s, cDC1s, and monocytes with anti-TNF-α mAb staining (left panels) and with its isotype control staining (right panels).
