# Supplementary material for: Development of an Inflammatory CD14+ Dendritic Cell Subset in Humanized Mice
Source: Front Immunol. 2021 Mar 15;12:643040. doi: 10.3389/fimmu.2021.643040 (PMC8005643; doi:10.3389/fimmu.2021.643040)
Supplement: Supplementary Table 1 — List of hNOJ mice used in the present study. [file Table_1.pdf]

Supplementary Table 1. List of hNOJ mice used in the present study.

Mouse ID	Donor ID	IVT status	% chimerism (at wk-old)*	Wk-old used in the experiment(s)	Data presentation (Figure)
G421-f1		-	15.9 (wk 12)	wk 15	S2E
G421-f2	180926-2	-	13.7 (wk 12)	wk 15	S2E
G421-m1		-	5.6 (wk 12)	wk 15	S2E
G448-f1		FLT3L+GM-CSF	17.1 (wk 12)	wk 16	
G448-f2		FLT3L+GM-CSF	18.7 (wk 12)	wk 16	
G448-f3	190404-2	FLT3L+GM-CSF	20.1 (wk 12)	wk 16	3A, 3B, 3C, 3D, S4 (D1 sample)
G448-f4		FLT3L+GM-CSF	19.8 (wk 12)	wk 16	
G448-m1		FLT3L+GM-CSF	4.9 (wk 12)	wk 16	
G449-f1		FLT3L+GM-CSF	9.3 (wk 12)	wk 15	
G449-f2		FLT3L+GM-CSF	19.0 (wk 12)	wk 15	
G449-f3	190411-1	FLT3L+GM-CSF	15.5 (wk 12)	wk 15	3A, 3B, 3C, 3D, S4 (D2 sample)
G449-f4		FLT3L+GM-CSF	14.6 (wk 12)	wk 15	
G449-m2		FLT3L+GM-CSF	5.0 (wk 12)	wk 15	
G449-m1		FLT3L+GM-CSF	3.9 (wk 12)	wk 15	1D
G450-f1		FLT3L+GM-CSF	27.4 (wk 12)	wk 16	
G450-m1		FLT3L+GM-CSF	20.4 (wk 12)	wk 16	
G450-m2	190411-2	FLT3L+GM-CSF	31.2 (wk 12)	wk 16	3A, 3B, 3C, 3D, S4 (D3 sample)
G450-m4		FLT3L+GM-CSF	24.8 (wk 12)	wk 16	
G450-m5		FLT3L+GM-CSF	20.6 (wk 12)	wk 16	
G450-m3		FLT3L+GM-CSF	11.6 (wk 12)	wk 16	1D, 1E (Donor-1)
G461-f2	190826-1	-	25.6 (wk 12)	wk 17	S2E
G461-f4		-	25.8 (wk 12)	wk 17	S2E
G469-f1		FLT3L+GM-CSF	21.0 (wk 12)	wk 16	4A, 4C, 4D, 4E, 4F (Mock)
G469-f2	191122-2	FLT3L+GM-CSF	18.2 (wk 12)	wk 16	4A, 4C, 4D, 4E, 4F (LPS)
G469-f4		FLT3L+GM-CSF	15.8 (wk 12)	wk 16	4A, 4B, 4C, 4D, 4E, 4F, S5B (LPS)
G469-f5		FLT3L+GM-CSF	4.6 (wk 12)	wk 16	4A, 4C, 4D, 4E, 4F (Mock)
G470-f1		FLT3L+GM-CSF	9.9 (wk 12)	wk 15	4A, 4C, 4D, 4E, 4F (Mock)
G470-f2	200110-1	FLT3L+GM-CSF	17.2 (wk 12)	wk 15	4A, 4C, 4D, 4E, 4F (Mock)
G470-f4		FLT3L+GM-CSF	16.0 (wk 12)	wk 15	4A, 4B, 4C, 4D, 4E, 4F (LPS)
G471-f2	200225-2	FLT3L+GM-CSF	7.9 (wk 12)	wk 15	4A, 4B, 4C, 4D, 4E, 4F, S5A (LPS)
G473-f1		FLT3L+GM-CSF	23.1 (wk 12)	wk 15	1B, 1C, 1D, 1E, 2C, 2E, S2D, S2E, S3B, S3D (Donor-2)
G473-f2		FLT3L+GM-CSF	27.3 (wk 12)	wk 15	1B, 1C, 1D, 1E, 2C, 2E, S2D, S2E, S3B, S3D (Donor-2)
G473-m1	200210-1	FLT3L+GM-CSF	7.9 (wk 12)	wk 15	1B, 1C, 1D, 1E, 2C, 2E, S2D, S2E, S3B, S3D (Donor-2)
G473-m2		FLT3L+GM-CSF	14.5 (wk 12)	wk 15	1B, 1C, 1D, 1E, 2C, 2E, S2D, S2E, S3B, S3D (Donor-2)
G473-m3		FLT3L+GM-CSF	5.5 (wk 12)	wk 15	1B, 1C, 1D, 1E, 2C, 2E, S2D, S2E, S3B, S3D (Donor-2)
G474-f1		FLT3L+GM-CSF	11.9 (wk 12)	wk 15	1B, 1C, 1D, 1E, 2A, 2B, 2C, 2D, 2E, S2D, S2E, S3B, S3C, S3D (Donor-3)
G474-f2	200210-2	FLT3L+GM-CSF	22.4 (wk 12)	wk 15	1B, 1C, 1D, 1E, 2A, 2B, 2C, 2D, 2E, S2D, S2E, S3B, S3C, S3D (Donor-3)
G474-f3		FLT3L+GM-CSF	13.7 (wk 12)	wk 15	1B, 1C, 1D, 1E, 2A, 2B, 2C, 2D, 2E, S2D, S2E, S3B, S3C, S3D (Donor-3)
G474-m1		FLT3L+GM-CSF	5.7 (wk 12)	wk 15	1B, 1C, 1D, 1E, 2A, 2B, 2C, 2D, 2E, S2D, S2E, S3B, S3C, S3D (Donor-3)
G475-f1	200217-2	FLT3L+GM-CSF	7.7 (wk 12)	wk 15	4A, 4C, 4D, 4E, 4F (Mock)
G476-f1	200221-1	FLT3L+GM-CSF	5.7 (wk 12)	wk 15	4A, 4B, 4C, 4D, 4E, 4F (LPS)
G478-f1		FLT3L+GM-CSF	29 (wk 15)	wk 15	1B, 1C, 1D, 1E, 2C, 2E, S2D, S2E, S3A, S3B, S3D (Donor-4)
G478-f2	180307-1	FLT3L+GM-CSF	33.8 (wk 15)	wk 15	1B, 1C, 1D, 1E, 2C, 2E, S2D, S2E, S3A, S3B, S3D (Donor-4)
G478-f3		FLT3L+GM-CSF	19.5 (wk 15)	wk 15	1B, 1C, 1D[#], 1E, 2C, 2E, S2D, S2E, S3A, S3B, S3D (Donor-4)
G479-m2		FLT3L+GM-CSF	3.4 (wk 12)	wk 15	1B, 1C, 1D[#], 1E, S2D, S2E (Donor-5)
G479-m3	180402-2	FLT3L+GM-CSF	6.3 (wk 12)	wk 15	1D, 1E (Donor-5)
G479-m4		FLT3L+GM-CSF	6.6 (wk 12)	wk 15	1A, 1B, 1C, 1D[#], 1E, S1A, S1B, S2D, S2E (Donor-5)
G481-m1	180522-2	FLT3L+GM-CSF	17.3 (wk 12)	wk 15	1B, 1C, 1D, S2D, S2E
G482-m2	200225-1	FLT3L+GM-CSF	4.6 (wk 12)	wk 16	1D, 1E (Donor-6)
G482-m3		FLT3L+GM-CSF	6.5 (wk 12)	wk 16	1D, 1E (Donor-6)
G483-m3	200204-1	FLT3L+GM-CSF	5.4 (wk 12)	wk 16	1D, 1E (Donor-7)
G485-f1		FLT3L+GM-CSF	9.8 (wk 12)	wk 16	1D, 1E (Donor-8)
G485-f2	191031-1	FLT3L+GM-CSF	12.1 (wk 12)	wk 16	1D, 1E (Donor-8)
G485-f3		FLT3L+GM-CSF	23.1 (wk 12)	wk 16	1D, 1E (Donor-8)
G487-m1	200825-2	FLT3L+GM-CSF	11.3 (wk 12)	wk 16	1D, 1E (Donor-9)
G488-f1		FLT3L+GM-CSF	49.7 (wk 12)	wk 16	1D, 1E (Donor-10)
G488-m1	200908-2	FLT3L+GM-CSF	22.1 (wk 12)	wk 16	1D[#], 1E, S1D (Donor-10)
G488-m2		FLT3L+GM-CSF	10.0 (wk 12)	wk 16	1D, 1E (Donor-10)
			* % human CD45+ cells within total PB cells		[#] Representative histogram
