# Supplementary material for: Development of an Inflammatory CD14+ Dendritic Cell Subset in Humanized Mice
Source: Front Immunol. 2021 Mar 15;12:643040. doi: 10.3389/fimmu.2021.643040 (PMC8005643; doi:10.3389/fimmu.2021.643040)
Supplement: Supplementary Table 2 — Gene signatures used in GSEA analysis. [file Table_2.pdf]

Supplementary Table 2. Gene signatures used in GSEA analysis.

cDC2>ALL	DC3>ALL	cDC2>DC3	DC3>DC2
CD1C	S100A9	HLA-DPB1	BACH1
FCER1A	S100A8	HLA-DQB1	CA5BP1
CLEC10A	VCAN	HLA-DQA1	TSC2
ADAM8	LYZ	HLA-DQA2	SHOC2
CD1D	ANXA1	CD1C	HPCAL1
FCGR2B	PLBD1	HLA-DOB	PVR
CLEC4A	RNASE2	P2RY14	RIPK2
SLC2A3	FCER1A	ARL4C	STIM1
CD33	SLC2A3	CLIC2	ID1
ETS2	CD163	CLEC17A	IKBKE
CLIC2	CSF3R	C10ORF128	KCNN4
PEA15	MNDA	CALHM6	EMP1
CACNA2D3	CD14	ASAP1	PLPPR2
CD1E	NAIP	SLC41A2	GPBAR1
MBOAT7	CSTA	SLAMF7	MKNK1
C10ORF128	FCN1	CST7	KIAA0513
NR4A2	CD1D	PKIB	FOXO3
GPAT3	FPR1	HSPA7	EMC3
ENTPD1	F13A1	CXCL16	YWHAG
CD2	CLEC10A	RUNX3	TAB1
PER1	CES1	WDFY4	OSM
PID1	PID1	IL18R1	GABARAPL1
AREG	S100A12	FCGR2B	ASPH
PTGS1	MTMR11	MYO1E	PDLIM7
SMN1	SMN1	AXL	QPCT
CLEC17A	LAT2	PEA15	RIN2
ITGA5	RETN	SIGLEC10	MRPS23
CREB5	TMEM173	CD1E	PLXND1
PTAFR	AOAH	GOLGA8B	CLEC12A
NOD2	RAB3D	IFITM1	TMEM176A
CCR6	CD36	ITGB2-AS1	PISD
	MGST1	FEZ1	PLA2G7
	TREM1	INSIG1	TMEM141
	HNMT	SPATS2L	NINJ1
	CES1P1	GRIP1	AGTRAP
	ADAM15	MCOLN2	BLVRA
	IL13RA1	SERTAD3	HBEGF
	MICAL2	PPP1R14A	DMXL2
	ITGA5	UVRAG	CARD19
	CREB5	SIGLEC6	IL1B
	IL1B	KPNA6	NLRP12
	NR4A2	LGMN	SORL1
	MPP7	SPIB	NFE2
	PTAFR	SNRPN	ADAM15
	HBEGF	WFDC21P	CCDC69
	NFE2	TOP1MT	SULT1A1
	ASGR1		TOM1
	BST1		KCNE3
	IL1RN		PYGL
	NOD2		SLC11A1
	NLRP3		HK3
	LMNA		ACSL1
	CARD19		IER3
	IL27RA		CFD
	NLRP12		LMNA
	RAB27A		MSRB1
	EREG		TREM1
			PILRA
			ASGR1
			TXNRD1
			GLUL
			PSTPIP1
			CSF3R
			STAB1
			RETN
			SERPINA1
			SLC7A7
			CTSD
			NEAT1
			CES1P1
			FPR1
			CD163
			S100A12
			CYBB
			F13A1
			CES1
			BST1
			MTMR11
			CD36
			MGST1
			RAB3D
			PLBD1
			TMEM176B
			CD14
			FCN1
			RNASE2
			VCAN
			S100A8
			S100A9

Note: Discriminative genes for each subset reported by Villani *et al.*
