# Supplementary material for: Development of an Inflammatory CD14+ Dendritic Cell Subset in Humanized Mice
Source: Front Immunol. 2021 Mar 15;12:643040. doi: 10.3389/fimmu.2021.643040 (PMC8005643; doi:10.3389/fimmu.2021.643040)
Supplement: Supplementary Figure 1 — Flow cytometric gate setting of human DC and monocyte populations in the present study. Cells were prepared from the spleen of hNOJ mice following IVT and human peripheral blood. (A) A representative gating strategy for CD141+ population, CD1c+ population, and CD1c−CD141− (DN) population of hNOJ mice. (B) Representative flow cytometry profiles for cDC1s, cDC2s, CD14+CD1c+ cells, and CD14high monocytes with anti-CD14 monoclonal antibody (mAb) staining (upper panels) and with its isotype control staining (lower panels). (C) A representative flow cytometry profile for CD14highCD16− classical monocytes in DN population of human PBMCs under the same staining condition of hNOJ mice samples. (D) Representative flow cytometry profiles for CD14high and CD14low cells in DN population of hNOJ mice. The histogram shows the PE-fluorescence intensity of CD14high cells (red: anti-CD88 mAb staining, orange: isotype control staining) and CD14low cells (blue: anti-CD88 mAb staining, green: isotype control staining). [file Image_1.pdf]

Supplementary Figure 1

Iwabuchi et al

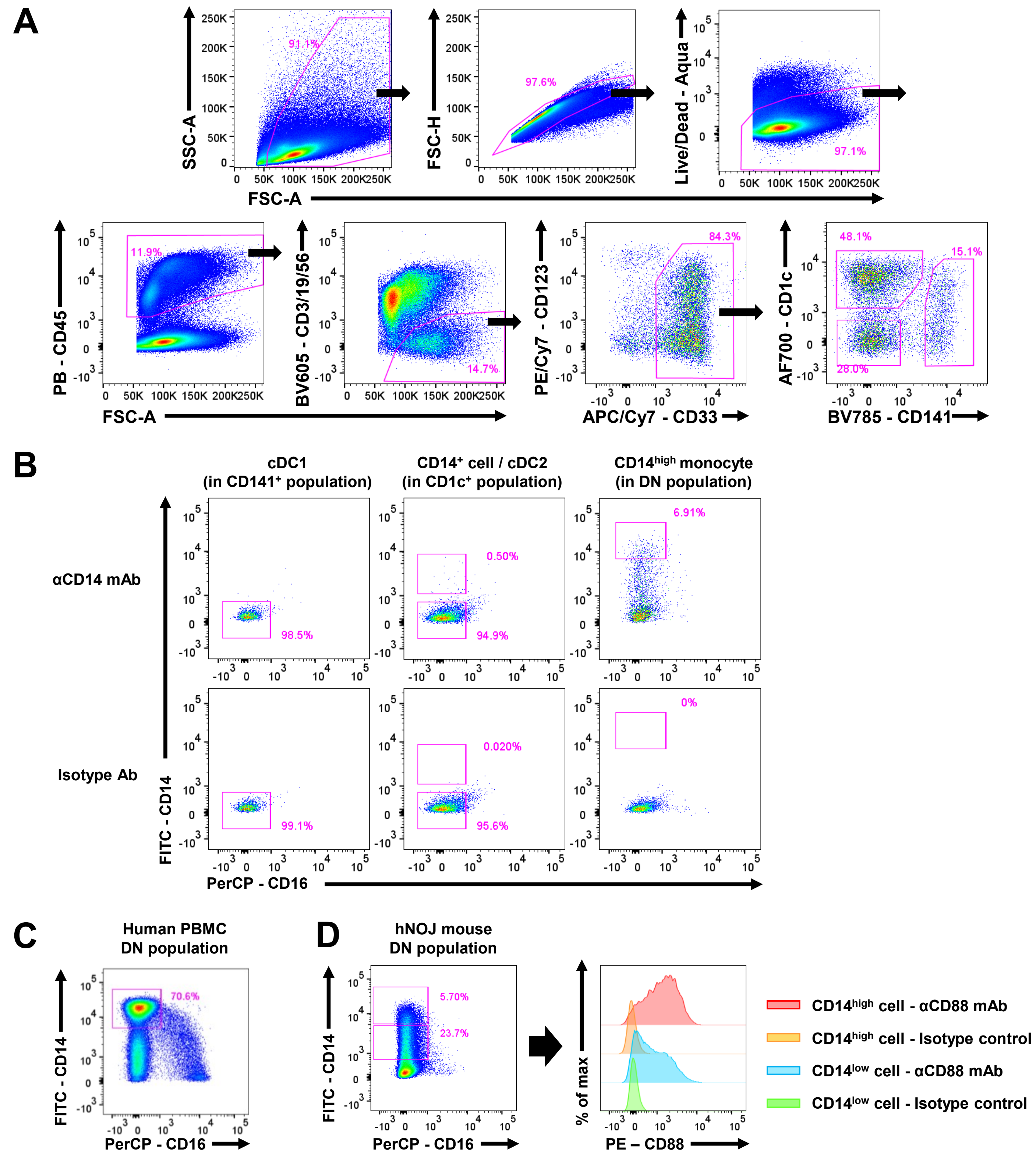

Supplementary Figure 1. Flow cytometric gate setting of human DC and monocyte populations in the present study. Cells were prepared from the spleen of hNOJ mice following *in vivo* transfection (IVT) and human peripheral blood. **(A)** A representative gating strategy for CD141^{+} population, CD1c^{+} population, and CD1c^{-}CD141^{-} (DN) population of hNOJ mice. **(B)** Representative flow cytometry profiles for cDC1s, cDC2s, CD14^{+}CD1c^{+} cells, and CD14^{high} monocytes with anti-CD14 monoclonal antibody (mAb) staining (upper panels) and with its isotype control staining (lower panels). **(C)** A representative flow cytometry profile for CD14^{high}CD16^{-} classical monocytes in DN population of human PBMCs under the same staining condition of hNOJ mice samples. **(D)** Representative flow cytometry profiles for CD14^{high} and CD14^{low} cells in DN population of hNOJ mice. The histogram shows the PE-fluorescence intensity of CD14^{high} cells (red: anti-CD88 mAb staining, orange: isotype control staining) and CD14^{low} cells (blue: anti-CD88 mAb staining, green: isotype control staining).

Supplementary Figure 2

Iwabuchi et al

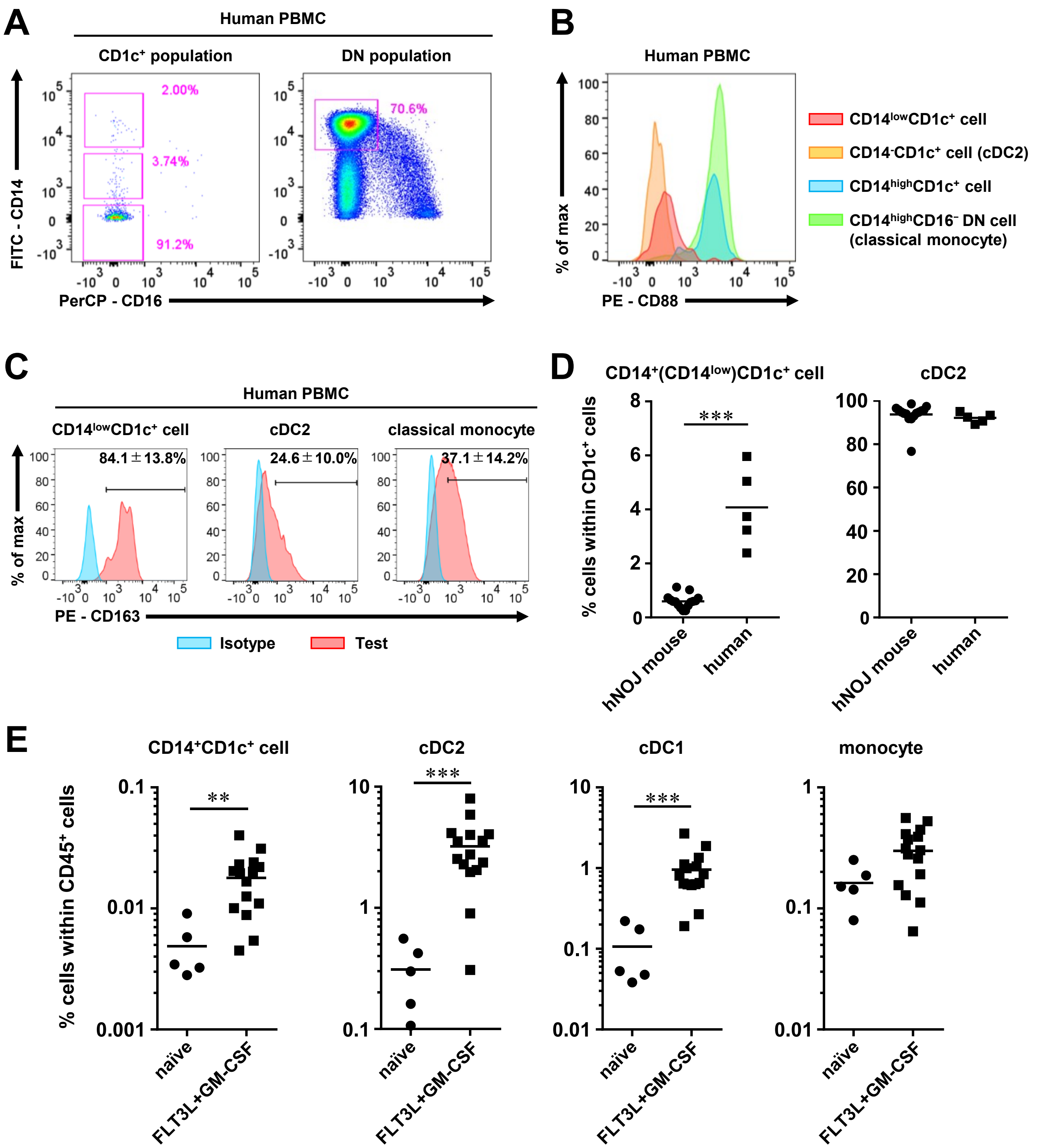

Supplementary Figure 2. Characterization of human DC and monocyte populations in humans and hNOJ mice.
Cells were prepared from human peripheral blood and the spleen of naïve hNOJ mice or hNOJ mice following IVT. **(A)** Representative flow cytometry profiles for CD1c$^+$ population and DN population of human PBMCs. **(B)** A representative histogram profile of CD88 expression on CD1c$^+$ population and DN population of human PBMCs [red: CD14^{low}CD1c$^+$ cells, orange: CD14$^-$CD1c$^+$ cells (cDC2), blue: CD14^{high}CD1c$^+$ cells, CD14^{high}CD16$^-$ DN cells (classical monocyte)]. **(C)** Representative histogram profiles of CD163 expression on CD14^{low}CD1c$^+$ cells, cDC2s, and classical monocytes in human PBMCs (red: test marker staining, blue: isotype control staining). The percentages in each panel show the mean ± SD of marker positive cells in each population (n = 5). **(D)** Individual percentages of CD14$^+$(CD14^{low})CD1c$^+$ cells and cDC2s within CD1c$^+$ population in hNOJ mice (n = 15) and humans (n = 5). A significant difference (***P < 0.001) was determined using the Mann-Whitney U test. **(E)** Individual percentages of CD14$^+$CD1c$^+$ cells, cDC2s, cDC1s, and monocytes within human CD45$^+$ cells in naïve hNOJ mice (n = 5) and IVT-hNOJ mice (n = 15). Significant differences (**P < 0.01, ***P < 0.001) were determined using the Mann-Whitney U test.

Supplementary Figure 3

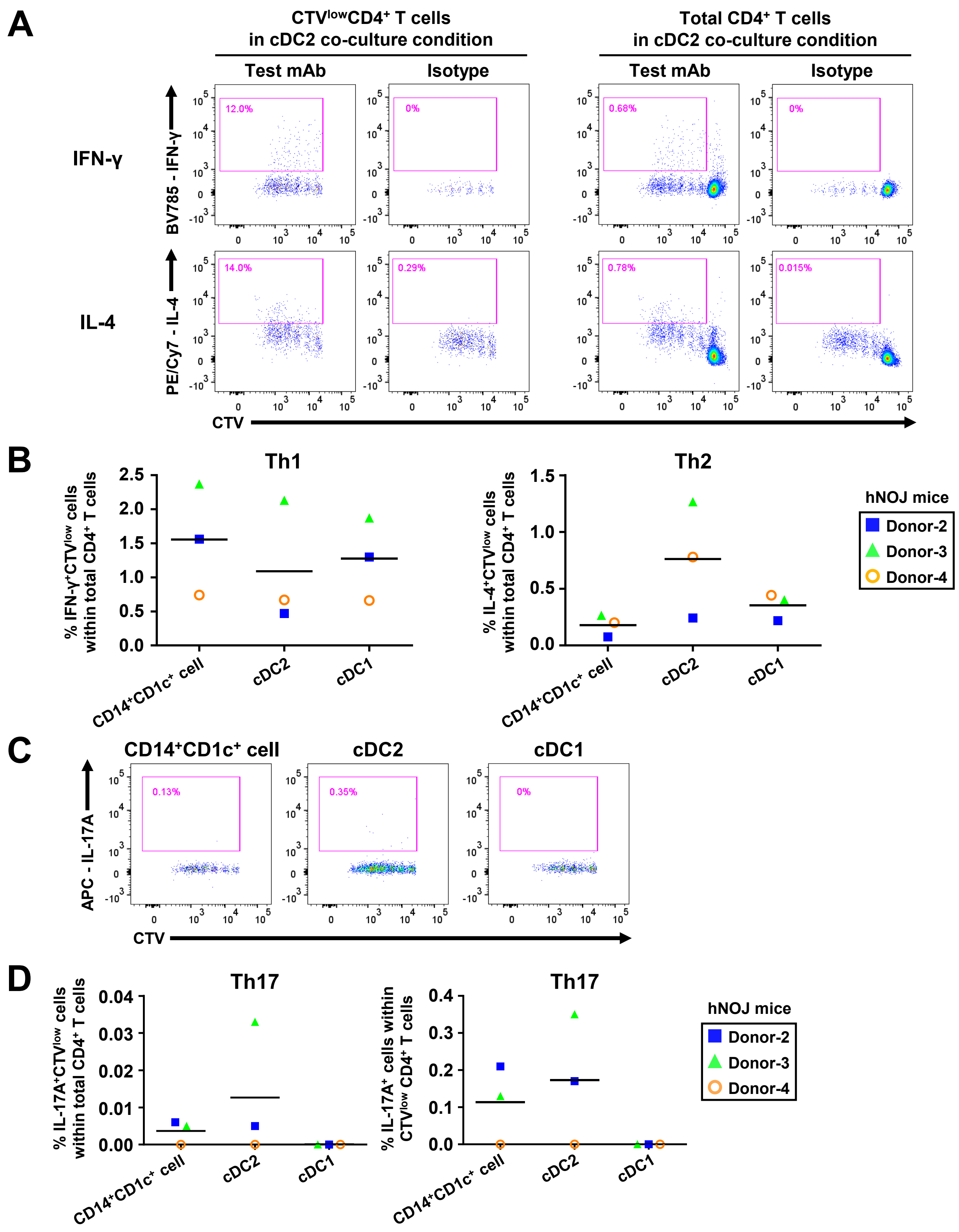

Supplementary Figure 3. Expression of IFN-γ, IL-4, and IL-17A in CD4⁺ T cells co-cultured with human DC subsets. **(A)** Gating strategy for IFN-γ⁺ cells (upper panels) and IL-4⁺ cells (lower panels) using identical cDC2 co-cultured CD4⁺ T cell samples stained with test and isotype antibodies. **(B)** Individual percentages of IFN-γ⁺CTV^{low} cells (Th1; n = 3) and IL-4⁺CTV^{low} cells (Th2; n = 3) within total CD4⁺ T cells. The Same color symbols show the same donor-derived hNOJ mice. The repeated-measures one-way ANOVA followed by the Holm-Sidak's multiple comparison test was used, and no significant differences were observed. **(C)** Representative flow cytometry profiles of IL-17A⁺ cells within CD4⁺ T cells. **(D)** Individual percentages of IL-17A⁺CTV^{low} cells within total CD4⁺ T cells and IL-17A⁺ cells within CTV^{low}CD4⁺ T cells. The same color symbols show the same donor-derived hNOJ mice. The repeated-measures one-way ANOVA followed by the Holm-Sidak's multiple comparison test was used, and no significant differences were observed.

Supplementary Figure 4

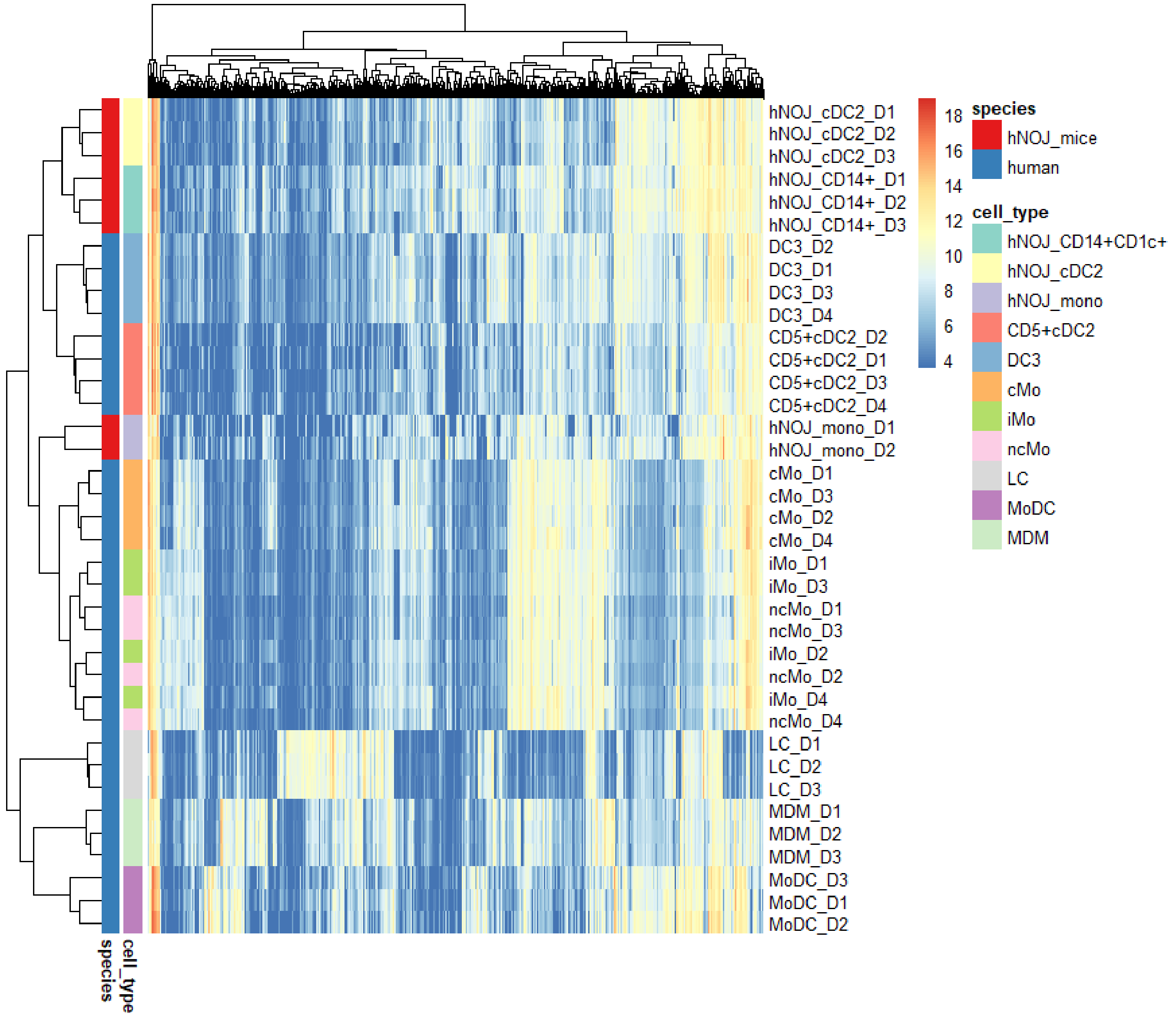

Supplementary Figure 4. Hierarchical clustering analysis among CD1c⁺ DC subsets and monocyte-related subsets in hNOJ mice and humans.

Heatmap visualization of the z-scores for the 1000 most variable genes among hNOJ mice samples [CD14⁺CD1c⁺ cell (n = 3), cDC2 (n = 3), and monocyte (n = 2)] and human samples [CD5⁺ cDC2 (n = 4), DC3 (n = 4), classical monocyte (cMo; n = 4), intermediate monocyte (iMo; n = 4), non-classical monocyte (ncMo; n = 4), monocyte-derived DC (MoDC; n = 3), monocyte-derived macrophage (MDM; n = 3), and Langerhans cell (LC, n = 3)] using the hierarchical clustering analysis.

Supplementary Figure 5

Iwabuchi et al

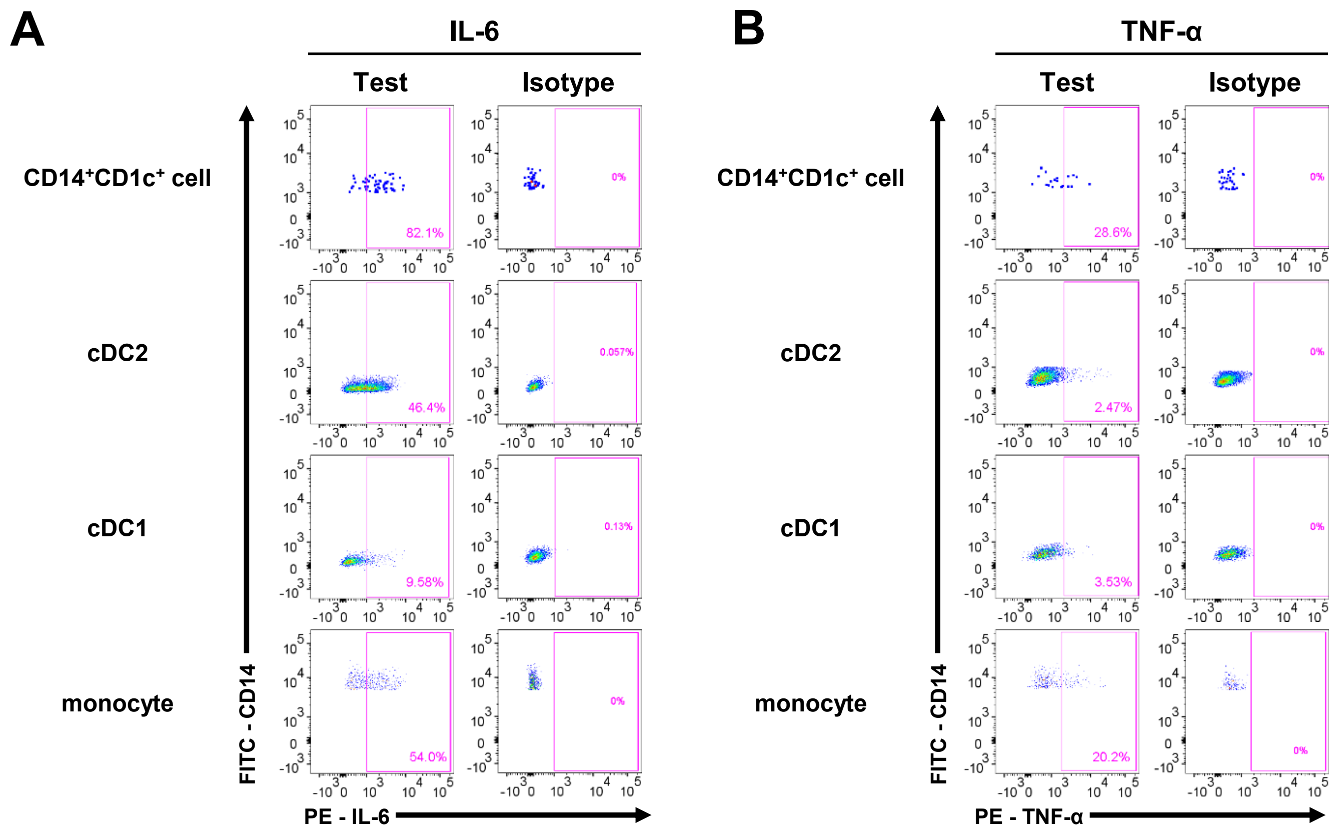

Supplementary Figure 5. Flow cytometric gate setting of IL-6$^+$ cells and TNF-α$^+$ cells in cell subsets.
Cells were prepared from the spleen of IVT-hNOJ mice following intraperitoneal LPS injection. **(A)** Representative flow cytometry profiles for CD14$^+$CD1c$^+$ cells, cDC2s, cDC1s, and monocytes with anti-IL-6 mAb staining (left panels) and with its isotype control staining (right panels). **(B)** Representative flow cytometry profiles for CD14$^+$CD1c$^+$ cells, cDC2s, cDC1s, and monocytes with anti-TNF-α mAb staining (left panels) and with its isotype control staining (right panels).
